# Human infections with Eurasian avian-like swine influenza virus detected by coincidence via routine respiratory surveillance systems, the Netherlands, 2020 to 2023

**DOI:** 10.2807/1560-7917.ES.2025.30.19.2400662

**Published:** 2025-05-15

**Authors:** Dirk Eggink, Annelies Kroneman, Jozef Dingemans, Gabriel Goderski, Sharon van den Brink, Mariam Bagheri, Pascal Lexmond, Mark Pronk, Erhard van der Vries, Evelien Germeraad, Diederik Brandwagt, Manon Houben, Mariëtte van Hooiveld, Joke van der Giessen, Rianne van Gageldonk-Lafeber, Ron Fouchier, Adam Meijer

**Affiliations:** 1 Centre for Infectious Disease Control, National Institute for Public Health and Environment (RIVM), Bilthoven, the Netherlands; 2 Department of Medical Microbiology, Infectious Diseases & Infection Prevention, Care and Public Health Research Institute (CAPHRI), Maastricht University Medical Center, Maastricht, the Netherlands; 3 Department of Viroscience, Erasmus Medical Center (EMC), Rotterdam, the Netherlands; 4 Wageningen Bioveterinary Research (WBVR), Lelystad, the Netherlands; 5 Royal GD, Deventer, the Netherlands; 6 Nivel, Utrecht, the Netherlands

**Keywords:** swine influenza virus, zoonoses, human surveillance, respiratory infections

## Abstract

**Background:**

Zoonotic influenza, including infections with avian and swine influenza A viruses (swIAV), is a notifiable disease in the Netherlands. Human cases infected with swIAV have previously been rarely detected in the Netherlands.

**Aim:**

We aimed to describe detection and characterisation of Eurasian avian-like swIAV infections in humans in the Netherlands 2020–2023.

**Methods:**

The Dutch National Influenza Center coordinates different activities to monitor respiratory infections and circulating human influenza viruses. This monitoring includes sentinel surveillance in general practitioner practices, community participatory surveillance and characterisation of influenza viruses received from diagnostic laboratories. A subset of the specimens positive for influenza A virus from the monitoring activities are sent for further characterisation. We characterised swIAV from human patients using whole genome sequencing, tested the viruses for antiviral susceptibility and in haemagglutination inhibition assays for antigenic characterisation and compared them with previous detections from humans and pigs.

**Results:**

Avian-like swine influenza virus was detected in three persons presenting with mild respiratory symptoms, and all recovered fully. Only one patient had close contact with pigs shortly before the start of symptoms. Sequence analyses of the viruses showed clustering with swAIV from pigs in a recently initiated surveillance system on pig farms.

**Conclusions:**

These human cases show that swIAV viruses with zoonotic potential are enzootic in the Netherlands. Finding them by coincidence suggests human infections might occur more frequently than noticed.

**Key public health message**

**What did you want to address in this study and why?**

Human infections with avian or swine influenza viruses can cause severe disease. These viruses could possibly start circulating among humans, causing outbreaks or even a pandemic. We here describe human infections with swine influenza virus identified via different monitoring systems in the Netherlands.

**What have we learnt from this study?**

Human infections with swine influenza viruses can also present as mild disease which is hard to distinguish from other respiratory infections like the common cold or influenza. Especially as these are often not formally laboratory confirmed. In addition, there are no systems in place to specifically identify human infections with swine influenza virus. Therefore, infections might often remain undetected.

**What are the implications of your findings for public health?**

Human infections with swine influenza might occur more frequently than noticed in the regular disease diagnostics and monitoring. It is therefore difficult to assess the real risk for the general population.

## Introduction

Sporadic human infections with avian or swine influenza A virus (swIAV) have been reported. Zoonotic influenza, including human infections with avian influenza A virus or swIAV, is notifiable in the Netherlands and national and international guidelines state that local and national public health services need to be timely notified of laboratory-confirmed cases allowing source finding and contact tracing.

Swine influenza A viruses are genetically and antigenically distinct for different continents, due to introductions into pig populations from different origins and at different time points. Limited inter-continental spread between pigs or pig farms seems to occur [1-3]. In Europe, four swIAV haemagglutinin (HA) lineages are enzootic: H1 classical swine lineage (clade 1A) including H1pdm09-like viruses, H1 human seasonal lineage (clade 1B), H1 Eurasian avian-like lineage (clade 1C) and European human-like H3 lineage. These HA lineages are combined with four neuraminidase (NA) lineages: N1pdm09-like, avian N1 and two swine N2 lineages. Knowledge about circulating genotypes of swIAV is limited and difficult to obtain due to limited surveillance of swIAV on pig farms in most countries in Europe, including the Netherlands, especially after the European Surveillance Network for Influenza in Pigs (ESNIP 3) was terminated in 2013 [1].

Human infections with swIAV have been detected sporadically in Europe whereas in the United States (US), such infections have been detected more frequently [2,4-6]. These infections occurred mainly after close contact with infected pigs on agricultural fairs [5]. In addition, the A(H1N1)pdm09 pandemic in 2009 was highly likely caused by direct spillover from infected pigs and subsequent human-to-human transmission [7]. In the Netherlands, swIAV was detected in six persons 1986–2019 [4,8-13]. The previous detections of swIAV infections in humans in the Netherlands were mostly coincidental, in hospitalised persons presenting with severe disease [10], as no surveillance system to detect zoonotic spillovers from pigs to humans is in place with the aim to monitor specific risk groups upon exposure. This contrasts with monitoring of individuals exposed to poultry infected with highly pathogenic avian influenza A virus (HPAI) for which passive and active monitoring is in place in the Netherlands.

Here we describe detection of Eurasian swIAV infection in three persons during routine influenza surveillance activities in 2020, 2022 and 2023 in the Netherlands.

## Methods

### Influenza A surveillance in the Netherlands

The National Influenza Center (NIC) in the Netherlands, operating from the Dutch National Public Health Institute (RIVM), Erasmus Medical Center (EMC) and Nivel, coordinates several activities to monitor respiratory infections and circulating influenza viruses. This includes sentinel surveillance in primary care (Nivel Primary Care Database – Sentinel Practices) [14], community participatory surveillance (Infectieradar) [15] and subtyping and characterisation of representative clinical specimens positive for influenza virus from hospital and peripheral diagnostic laboratories or by request from these laboratories. In addition, the NIC assists clinical microbiology laboratories in questions regarding specificity and sensitivity of diagnostic methods, for example related to novel influenza virus subtypes or lineages [16].

The sentinel surveillance within Nivel Primary Care Database aims to cover a patient population which represents 1% of the national population. General practitioners (GPs) report patients with influenza-like illness (ILI) [14]. A nose swab and a throat swab combined in one tube of transport medium is obtained from a subset of patients with ILI or acute respiratory infection (ARI) by ca 40 (2020) to 140 (since 2022) practices spread throughout the Netherlands. All swabs are tested for a panel of respiratory viruses, including influenza A virus by generic influenza A virus specific PCR [11,17]. Primer sequences are presented in Supplementary Table S2.

The participatory surveillance Infectieradar monitors the prevalence of respiratory symptoms in the general population among 12,000–15,000 adult participants [15]. As of October 2022, each week, a random subset of 200 participants reporting respiratory symptoms that week [15], is asked to send a self-collected nose-throat swab, which is subsequently tested for a panel of respiratory viruses using the RespiFinder 2Smart 24-pathogen multiplex nucleic amplification assay (Pathofinder, Maastricht, the Netherlands), which includes influenza A virus.

From the network of diagnostic laboratories, which are mostly associated with hospitals and some peripheral laboratories, a maximum of five clinical specimens collected from their influenza virus positive patient population are submitted per laboratory and per week to the NIC for further characterisation.

A One Health monitoring was conducted (2022–2023) covering the genomic, antigenic and antiviral susceptibility profiling of swIAVs circulating in pigs in the Netherlands as described in detail elsewhere [18]. In addition, when a zoonotic transmission is suspected, national authorities take samples on animal farms as part of routine source and contact tracing.

### Characterisation of swine influenza A viruses

Specimens positive for influenza A virus from sentinel GP surveillance, participatory surveillance and the hospital and peripheral laboratories were further analysed by subtype-specific PCR and/or nanopore sequencing. Primer details for all diagnostic PCRs and whole genome sequencing (WGS) are described in Supplementary Table S2. Viral RNA was isolated using MagNA Pure 96 (MP96) (Roche, Basel, Switzerland) and amplified using a universal eight-segment PCR using UNI primers [19]. The PCR products were diluted to normalise the samples and further processed using the native barcoding and ligation sequencing kits (Oxford Nanopore, Oxford, the United Kingdom) followed by sequencing on a GridION Flow Cell (R10.4.1) platform (Oxford Nanopore). Base calling and demultiplexing were done using dorado (https://github.com/nanoporetech/dorado). Consensus sequences were obtained using the ViroConstrictor version 1.4 (https://github.com/RIVM-bioinformatics/ViroConstrictor). Phylogenetic trees were made using TreeViewer (https://treeviewer.org). Swine-derived influenza virus sequences from the recently established One Health influenza surveillance system [18] were included in phylogenetic analyses.

Viruses were isolated from clinical specimens on mixed Madin-Darby canine kidney (MDCK)-2,6-sialyltransferase (SIAT)/MDCK-I cell monolayers (RIVM) or MDCK-SIAT or humanised MDCK-hCK cell monolayers (EMC).

Virus isolates were further characterised phenotypically by NA inhibition assay and determination of the antiviral drug (oseltamivir and zanamivir) concentration needed to inhibit the NA enzyme activity by 50% (IC_50_) [20]. Obtained NA, polymerase acid protein (PA) and matrix protein 2 (M2) genes were analysed for amino acid changes previously associated with reduced susceptibility for NA inhibitors, baloxavir marboxil and adamantane drugs.

Isolates and relevant reference viruses mentioned in Supplementary Table 1 were tested in the haemagglutination inhibition (HI) assay using turkey erythrocytes and ferret antisera for antigenic characterisation as described [21].

## Results

### Influenza A surveillance in the Netherlands

**Figure 1:**
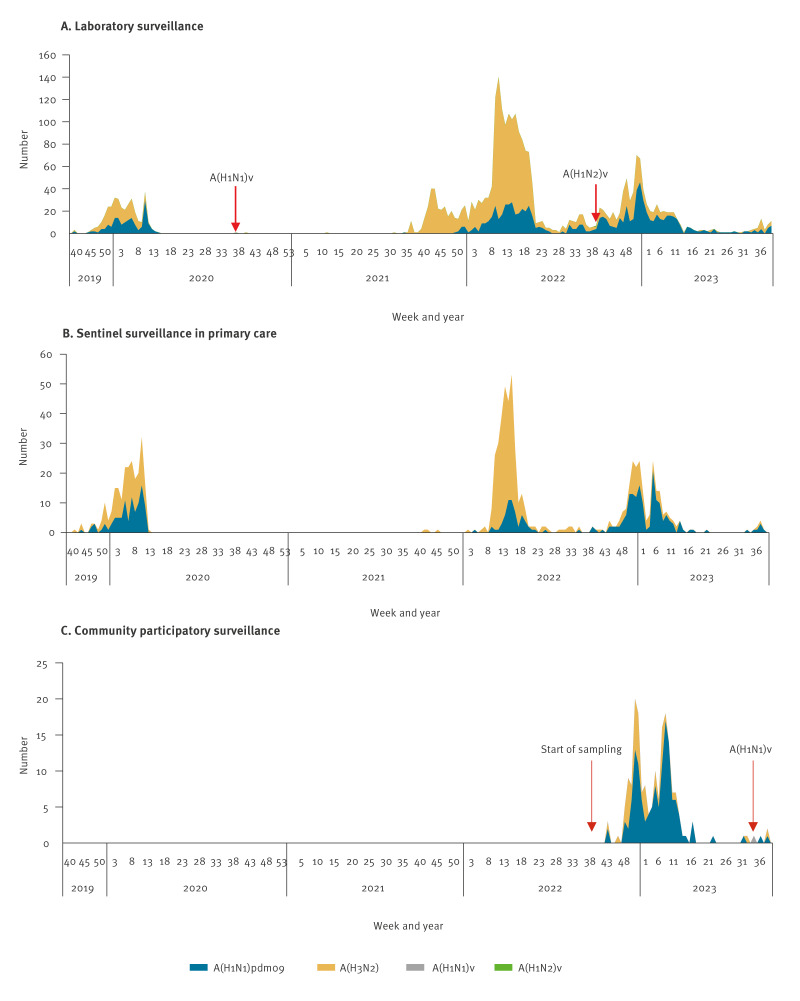
Subtyped influenza A viruses from humans within surveillance of influenza, the Netherlands, week 40 2019–week 39 2023 (n = 3,931)

A total of 3,931 influenza A virus positive specimens originating from sentinel surveillance in primary care, community participatory surveillance and representative influenza virus positive clinical specimens from hospital and peripheral diagnostic laboratories or by request from these laboratories, were successfully analysed week 40 2019–week 39 2023 (Figure 1). Virus subtype was determined by subtype-specific PCR or sequencing. Co-circulation of human seasonal A(H3N2) (n = 2,455) and A(H1N1)pdm09 (n = 1,473) viruses was seen for all respiratory seasons covered, although almost no influenza A viruses were detected between early 2020 and autumn 2021 as a consequence of non-pharmaceutical interventions like social distancing and lockdowns during the COVID-19 pandemic. During the 2019/20 season, there was no clear dominant influenza A virus subtype. During the peak of the influenza waves in 2021/22 and 2022/23, subtypes A(H3N2) and A(H1N1)pdm09 were more prevalent. Swine influenza A virus was detected in three persons: in September 2020, October 2022 and August 2023 (Figure 1).

H: haemagglutinin; N: neuraminidase.

### Case descriptions

#### Patient 1 – September 2020

In September 2020, a person in their 30–40s (Patient A) with weakened immune system was hospitalised for stem cell transplantation. Specimens of Patient A tested negative for influenza A virus by RT-qPCR in a routine screening for respiratory pathogens in the hospital. Seven days after the stem cell transplantation, Patient A developed ILI. One day after, influenza A virus was detected in a throat flush specimen by RT-qPCR. This virus was not subtyped at that time. Patient A was treated with oseltamivir for 5 days but still tested positive for influenza A virus 2 weeks after completion of this treatment. A specimen taken 23 days after symptom onset tested negative for influenza A virus. The patient recovered and was discharged in November 2020.

In November 2020, the hospital retrospectively used two specimens positive for influenza A virus, collected from Patient A, to validate the novel cobas liat SARS-CoV-2 and Influenza A/B molecular point-of-care test (mPOCT, cobas SARS-CoV-2 & Influenza A/B, the Cobas Liat System, Roche) assay. Both specimens, with quantification cycle (Cq) values 22 and 25 in the RT-qPCR for influenza A virus, were negative in the mPOCT assay. Therefore, the hospital submitted these two specimens to NIC/RIVM for characterisation to identify the reason for the negative mPOCT results. Regular molecular diagnostics for influenza A virus confirmed the PCR results initially obtained, but subtyping by PCR for human seasonal influenza virus and for avian H5 was negative. Whole genome sequencing characterised the virus as a Eurasian avian-like swine influenza A(H1N1)v virus clade 1C.2.1.

Patient A had no direct contact with pigs or other animals during the incubation period. Further source and contact tracing by the local municipal health service, including family members and hospital employees, did not provide any direct link to a likely source. No secondary infections were detected.

#### Patient B – October 2022

In October 2022, NIC/RIVM received an influenza A virus positive specimen from one of the clinical microbiology laboratories in the Netherlands as part of a national subset of specimens routinely analysed for subtype, genetic and antiviral drug susceptibility. This specimen originated from Patient B, a person in their 20–30s, without any underlying illness at the time of diagnosis, but an autologous stem cell transplantation in March 2021. Patient B was presented at an emergency department of a hospital in October 2022 with acute onset of chills and fever the same day. Three weeks before the onset of these symptoms, Patient B developed a cough that worsened 1 week before the hospital consultation. Patient B was not hospitalised and was sent home with antimicrobial treatment to recover. The fever was resolved 3 days later. At the consultation, a combined nose and throat swab was collected and submitted to a diagnostic laboratory. The test was positive for influenza virus type A. The diagnostic laboratory submitted the influenza virus positive specimen for routine seasonal influenza virus characterisation to NIC/RIVM. The specimen was confirmed positive for influenza virus type A by RT-qPCR (Cq: 21.1) but subtyping by PCR for human seasonal influenza A viruses and for avian H5, H7 and H9 was negative. Sequencing with WGS characterised the virus as a Eurasian avian-like swine influenza A(H1N2)v virus clade 1C.2. 2. Nose and throat specimens collected 16 days after the first specimen were still weakly positive for influenza A virus, but Patient B was symptom-free.

Patient B was working remotely as an administrative assistant for a pig farm. Patient B was invited for a work visit for 3 days to the farm and reported direct unprotected contact (cuddling) with piglets in the week before the hospital visit. As some of the respiratory symptoms started already 3 weeks before the hospital visit, another respiratory infection might have been present that was not diagnosed shortly after the start of the symptoms. When the specimen was tested for other viruses using the multiplex respiratory assay (RespiFinder 2Smart), the specimen was positive for influenza A virus, but also a weak positive signal for adenovirus was seen, which might have caused the cough in the preceding period. Some piglets on the farm displayed clinical signs of illness (sneezing), and seven nose/throat swabs were collected, of which five tested positive for swine influenza virus A(H1N2) and three specimens were cultured for subsequent WGS using the same methods as described by De Vries et al. [18].

Contact tracing and monitoring was conducted by the local municipal health service. One close contact was identified, who did not develop any symptoms. Healthcare personnel of the hospital in which Patient B was seen used personal protective equipment as Patient B was considered possibly carrying methicillin-resistant *Staphylococcus aureus* (MRSA).

#### Patient C – August 2023

In August 2023, NIC/RIVM received a specimen from a participant of the participatory surveillance Infectieradar. The participant (Patient C) developed relatively mild respiratory symptoms with acute onset of chills, sneeze, cough, headache and asthenia followed by development of fever the day after. Patient C reported no underlying disease. The specimen tested positive for influenza A virus by initial screening. Subtyping by PCR for human seasonal influenza A viruses and for avian H5, H7 and H9 was negative but WGS identified Eurasian avian-like swine influenza A(H1N1)v virus clade 1C.2.2. Patient C recovered shortly after and did not require any further healthcare.

Source and contact tracing by the local municipal health service did not identify any direct contact with pigs or pig farms. Patient C spent some time outdoors in an area close to pig farms. No contact persons with respiratory symptoms were identified, which could have been the source of the infection or secondary cases.

### Molecular characterisation

#### Genetic and phylogenetic analyses

**Figure 2:**
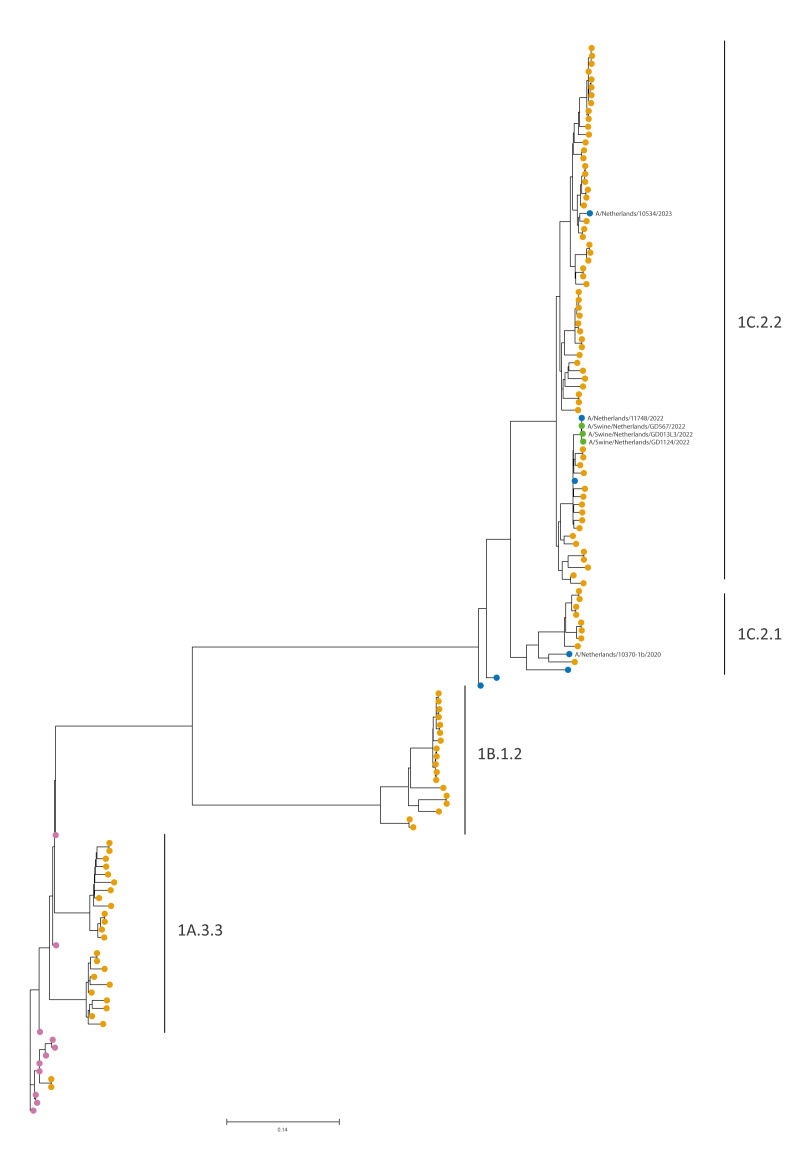
Phylogenetic tree of avian-like swine influenza A viruses from human patients (n = 3) and pigs (n = 116), the Netherlands, 2020–2023

HA: haemagglutinin; swIAV: avian-like swine influenza A virus.

Whole genome sequencing was successful for all segments for the viruses originating from the three patients (Table). The viruses from Patients A–C belonged to Eurasian avian-like swIAV with two viruses belonging to HA clade 1C.2.2 and one to the closely related 1C.2.1 clade (Figure 2). Viruses from two patients contained an NA of subtype N1 and one of N2. The internal genes of the three viruses resembled other Eurasian avian-like swIAV as presented in Supplementary Figures S1–8. These viruses shared no common genome segments with human influenza A(H1N1)pdm09 or A(H3N2) viruses.

**Table:** Characteristics of avian-like swine influenza A viruses, the Netherlands, 2020–2023 (n = 3)

ID	Strain name	Subtype	HI titre^a^	Oseltamivir		Zanamivir		Markers for reduced antiviral susceptibility^c^
				IC_50_ (nM)	Fold-change^b^	IC_50_ (nM)	Fold-change^b^	
Reference	A/California/7/2009	A(H1N1)pdm09	7,680	Not applicable				
Patient A	A/Netherlands/10370–1b/2020 EPI_ISL_717720	A(H1N1)v	960	0.20	0.34	0.61	1.04	M2-V27I, M2-S31N
Patient B	A/Netherlands/11748/2022 EPI_ISL_15348505	A(H1N2)v	1,920	0.39	1.73	3.60	4.29	M2-L26I, M2-V27A, M2-S31N
Patient C	A/Netherlands/10534/2023 EPI_ISL_18168180	A(H1N1)v	1,280	0.54	0.95	0.65	1.12	M2-S31N, NA-Y155H^d^

GP: general practitioner; HI: haemagglutination inhibition; IC_50_: half-maximal inhibitory concentration; ID: identification code; M2: matrix protein 2, markers for reduced sensitivity to M2 ion channel blockers, adamantanes; NA: neuraminidase, markers for reduced sensitivity to NA inhibitors, i.e. oseltamivir and zanamivir used in the Netherlands; PA: polymerase acid.

^a^ Haemagglutination inhibition assay using reference ferret antisera against A/California/7/2009.

^b^ Compared with median IC_50_ of N-subtype matching human seasonal influenza viruses A(H1N1)pdm09 (n = 99; median IC_50_ oseltamivir 0.57 nM, zanamivir 0.58 nM) or A(H3N2) (n = 142; median IC_50_ oseltamivir 0.23 nM, zanamivir 0.84 nM) from the GP sentinel surveillance tested phenotypically with collection dates week 40 2019–week 39 2023. Fold-change ≥ 10 is considered reduced inhibited and > 100 highly reduced inhibited [29].

^c^ Markers for reduced sensitivity to PA endonuclease inhibitor baloxavir marboxil not detected. Markers for reduced sensitivity to neuraminidase inhibitors not detected in specimens from Patient A and B.

^d^ NA-Y155H causes oseltamivir highly reduced inhibition in former human seasonal influenza A(H1N1) viruses, but not in A(H1N1)pdm09 viruses. The A(H1N1)v virus and swine A(H1N1) viruses with the same amino acid substitution appeared also normally inhibited by oseltamivir and zanamivir [18].

Human cases of swIAV infection from the Netherlands are depicted in blue, with the three cases described in this paper indicated by their strain names. Sequences from the recently established One Health influenza surveillance system in the Netherlands [18] are depicted in orange and representative human seasonal vaccine strains, both H1N1 before 2009 and A(H1N1)pdm09, are indicated in pink. Swine influenza virus HA clades are indicated: clade 1A H1 classical swine, including H1pdm09-like viruses; clade 1B H1 human seasonal lineage and clade 1C H1 Eurasian avian-like lineage. Sequences originating from the swAIV isolated from pigs related to the likely source of Patient B are depicted in green.

For Patient B, the likely source was identified, and three positive swabs from pigs of the pig farm Patient B visited were analysed by WGS, showing high similarity (99–100% nt identity for all eight segments) between the virus of Patient B and the three cell-cultured virus isolates from the specimens taken from the pig farm (Figure 2), confirming this as the likely source for the human infection.

For Patient A and C, no direct link to (infected) pigs could be made. Patient A diseased in September 2020 before the monitoring for swIAV in pigs was started in the Netherlands. Due to the lack of swine influenza surveillance in the Netherlands at the time the first case occurred, it was not possible to properly analyse whether Patient A was infected by viruses circulating concurrently at pig farms or that specific genetic changes were present that could possibly indicate adaptation to humans. However, in 2022–2023, a One Health swine influenza monitoring project was set up in the Netherlands aiming to improve insights in circulating swine influenza virus genotypes and allowing comparison to human cases [18]. This allowed more detailed comparisons of the three cases, including the two for which no source was apparent. All genome segments from the viruses from Patients A–C clustered with recent swine-derived influenza virus sequences (Figure 2), indicating that the human cases were infected with swIAV enzootic in the Netherlands. More details can be seen in Supplementary Figures S1–7.

### Antiviral susceptibility

**Figure 3:**
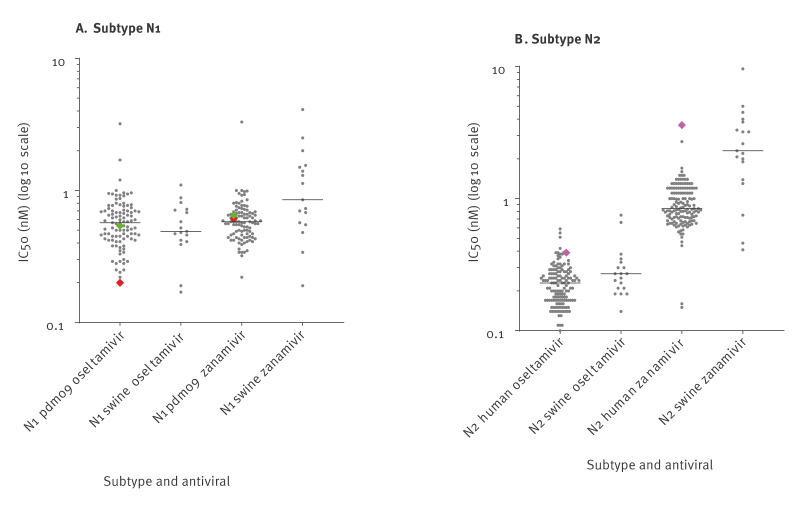
Antiviral sensitivity of avian-like swine influenza A viruses isolated from humans and pigs compared to human seasonal influenza viruses, the Netherlands, 2020–2023

IC50: half-maximal inhibitory concentration; N: neuraminidase.

Virus isolates of all three patients were tested for oseltamivir and zanamivir inhibition and compared to representative human seasonal influenza viruses (H1N1pdm20: n = 99 and H3N2: n = 142) and swine influenza viruses from the One Health swine influenza monitoring project (H1N1: n = 17 and H1N2: n = 19) (Figure 3).

A total of 277 viruses were included in the analysis. Reference human and swine influenza A viruses are represented as grey dots [18], whereas the viruses originating from the human cases are displayed in diamond symbols. Red: A/Netherlands/10370–1b/2020 H1N1v; green: A/Netherlands/10534/2023 H1N1v and magenta: A/Netherlands/11748/2022 H1N2v.

Whole genome sequencing of the virus from Patient A revealed the emergence of the NA-H275Y amino acid substitution previously associated with highly reduced inhibition by oseltamivir after the aforementioned oseltamivir treatment. This amino acid substitution was absent before treatment of Patient A started and present in ca 80% of the virus sequences in the specimen 5 days after completion of the oseltamivir course, but almost completely lost again 8 days after completion of the course. Following these results, two earlier and one later collected specimen from the patient positive for influenza A virus, were sent by the original hospital for characterisation. Whole genome sequencing revealed the same virus, and both specimens collected before the start of oseltamivir therapy appeared sensitive with residue NA-275H and no other amino acid substitutions indicating reduced inhibition by oseltamivir. In addition, virus with NA-275H from a specimen of patient A collected at the start of the oseltamivir therapy was isolated and displayed phenotypically normal inhibition by oseltamivir and zanamivir (Figure 3) [18]. Unfortunately, we were not successful in isolating virus with resistance marker NA-H275Y for phenotypic testing. For viruses from Patients B and C not treated with antivirals, no amino acid substitutions associated with reduced inhibition by oseltamivir or zanamivir were found (Table), and phenotypic testing revealed a normal inhibited phenotype for these antivirals (Figure 3).

Genetic markers indicative for resistance to M2 inhibitors were present in viruses from all three patients (e.g. M2-S31N), but no markers in PA for baloxavir reduced susceptibility were identified (Table).

### Antigenic characterisation

To investigate antigenic properties and possible cross-reactivity of A(H1N1)pdm09 antibodies, we performed haemagglutination inhibition assays using reference ferret antisera (Table). More details are presented in Supplementary Table S1. One of two swIAV clade 1A sera as well as clade 1A A(H1N1)pdm09 California/7/09 serum and most 1C sera reacted with the swIAV viruses from Patients A–C, showing that their HAs were antigenically related and similar to the previous zoonotic case A/Netherlands/Gent-193/19, but distinct from earlier Dutch zoonotic cases from 1986, 1993 and 2016 as shown in Supplementary Table 1.

## Discussion

We here describe the accidental detection of three infections with swIAV. Human infections with swIAV have been detected since the late 1950s [22]. Infections with swIAV are usually reported for people with direct or indirect exposure to pigs i.e. people visiting animal fairs or working on pig farms [5,23]. A total of 178 human infections with swIAV H1N1v, H1N2v and H3N2v globally were reported to the World Health Organization (WHO) and European Centre for Disease Prevention and Control (ECDC) between 2015 and 2022 of which 10 H1N1v and three H1N2v cases in Europe including two of the patients described here [24-26]. Interestingly, all viruses from the seven human swIAV cases from the Netherlands belonged to H1 clade 1C, similar to other geographical regions in the world, although other subclades and subtypes are also observed [4,27]. Influenza A viruses of these subtypes and clades are enzootic in western Europe and have been detected in pigs in Europe in recent years [1-3]. Whether clade 1C viruses pose a greater zoonotic risk warrants additional investigations. Nevertheless, the thorough source and contact tracing of the three patients did not identify additional human infections, showing that the genotype viruses involved may not have spread from human to human.

Viruses isolated from the three patients were inhibited by ferret antiserum to A/California/7/09, indicating some cross-reactivity of antibodies elicited by A(H1N1)pdm09-like virus infection or vaccines, although inhibition was reduced compared with inhibition of the homologous isolate. This suggests a possible level of antibody-mediated cross-reaction in the population; however, this needs to be studied in more detail using representative sera from the general population. Cellular immunity could also play a role but warrants additional analyses.

Interestingly, the three patients described here were detected via routine surveillance systems and NIC activities. The occurrence of these infections outside the traditional respiratory season is remarkable, although it is not possible at this point to draw conclusions about the risk of infection during or outside the respiratory season. In the Netherlands, there is no passive or active surveillance of humans exposed to pigs infected with swIAV, like farmers, their family members, veterinarians or individuals involved in transport or slaughtering of pigs. The experience in the US with swIAV exposure and human infections associated with visits to agricultural fairs suggests that close contact could be a major factor in zoonotic transmission, although the lack of direct exposure to (infected) pigs in two out of the three patients described, illustrates that other routes of transmission could exist. Due to the possible cross-reactivity of anti A(H1N1)pdm09-like antibodies, present in most, if not all, humans, with the zoonotic viruses, serological assays could not be used for source and contact tracing to investigate possible unnoticed human-to-human transmission. This likely results in underreporting of human infections with swIAV, in particular, as most patients with ILI in the general population are not sampled, let alone subtyped, especially those from mild to moderately severe cases that do not seek healthcare [28]. Therefore, (mild) human infections with swIAV could easily remain undetected. Undetected infections might result in human adaptation by adaptive mutations or reassortment with seasonal influenza viruses, which could increase risk for human-to-human transmission.

## Conclusion

Taken together, these three accidental swIAV detections in humans suggest that human infections with swIAV might occur more often than are currently reported and may remain undetected. Therefore, more data are needed for calculations of the burden of disease and for investigations of the zoonotic potential of swIAV for proper risk assessment.

## Data Availability

Generated human swIAV sequences are available via GISAID (https://gisaid.org/), accession numbers EPI_ISL_717720, EPI_ISL_15348505, EPI_ISL_18168180 and GISAID accession numbers EPI_ISL_19454939, EPI_ISL_19454940, EPI_ISL_19454941 for the related swine specimens.

## Supplementary Data

477000 Supplementary Material

## References

[1] Chepkwony S Parys A Vandoorn E Stadejek W Xie J King J Genetic and antigenic evolution of H1 swine influenza A viruses isolated in Belgium and the Netherlands from 2014 through 2019. Sci Rep. 2021;11(1):11276. 10.1038/s41598-021-90512-z 34050216 PMC8163766

[2] Lewis NS Russell CA Langat P Anderson TK Berger K Bielejec F The global antigenic diversity of swine influenza A viruses. eLife. 2016;5:e12217. 10.7554/eLife.12217 27113719 PMC4846380

[3] Watson SJ Langat P Reid SM Lam TT Cotten M Kelly M Molecular epidemiology and evolution of influenza viruses circulating within European swine between 2009 and 2013. J Virol. 2015;89(19):9920-31. 10.1128/JVI.00840-15 26202246 PMC4577897

[4] Freidl GS Meijer A de Bruin E de Nardi M Munoz O Capua I Influenza at the animal-human interface: a review of the literature for virological evidence of human infection with swine or avian influenza viruses other than A(H5N1). Euro Surveill. 2014;19(18):20793. 10.2807/1560-7917.ES2014.19.18.20793 24832117

[5] McBride DS Perofsky AC Nolting JM Nelson MI Bowman AS . Tracing the source of influenza A virus zoonoses in interconnected circuits of swine exhibitions. J Infect Dis. 2021;224(3):458-68. 10.1093/infdis/jiab122 33686399 PMC7989509

[6] Bowman AS Walia RR Nolting JM Vincent AL Killian ML Zentkovich MM Influenza A(H3N2) virus in swine at agricultural fairs and transmission to humans, Michigan and Ohio, USA, 2016. Emerg Infect Dis. 2017;23(9):1551-5. 10.3201/eid2309.170847 28820376 PMC5572863

[7] Garten RJ Davis CT Russell CA Shu B Lindstrom S Balish A Antigenic and genetic characteristics of swine-origin 2009 A(H1N1) influenza viruses circulating in humans. Science. 2009;325(5937):197-201. 10.1126/science.1176225 19465683 PMC3250984

[8] de Jong JC de Ronde-Verloop JM Bangma PJ van Kregten E Kerckhaert J Paccaud MF Isolation of swine-influenza-like A(H1N1) viruses from man in Europe, 1986. Lancet. 1986;2(8519):1329-30. 10.1016/S0140-6736(86)91450-9 2878189

[9] de Jong JC Paccaud MF de Ronde-Verloop FM Huffels NH Verwei C Weijers TF Isolation of swine-like influenza A(H1N1) viruses from man in Switzerland and The Netherlands. Ann Inst Pasteur Virol. 1988;139(4):429-37. 10.1016/S0769-2617(88)80078-9 3214596

[10] Fraaij PL Wildschut ED Houmes RJ Swaan CM Hoebe CJ de Jonge HC Severe acute respiratory infection caused by swine influenza virus in a child necessitating extracorporeal membrane oxygenation (ECMO), the Netherlands, October 2016. Euro Surveill. 2016;21(48):30416. 10.2807/1560-7917.ES.2016.21.48.30416 27934581 PMC5388114

[11] Meijer A Swaan CM Voerknecht M Jusic E van den Brink S Wijsman LA Case of seasonal reassortant A(H1N2) influenza virus infection, the Netherlands, March 2018. Euro Surveill. 2018;23(15):18-00160. 10.2807/1560-7917.ES.2018.23.15.18-00160 29667576 PMC6836195

[12] Parys A Vandoorn E King J Graaf A Pohlmann A Beer M Human Infection with Eurasian avian-like swine influenza A(H1N1) virus, the Netherlands, September 2019. Emerg Infect Dis. 2021;27(3):939-43. 10.3201/eid2703.201863 33622472 PMC7920694

[13] Rimmelzwaan GF de Jong JC Bestebroer TM van Loon AM Claas EC Fouchier RA Antigenic and genetic characterization of swine influenza A (H1N1) viruses isolated from pneumonia patients in The Netherlands. Virology. 2001;282(2):301-6. 10.1006/viro.2000.0810 11289812

[14] Donker GA. NIVEL Primary Care Database - Sentinel Practices 2015. NIVEL: Utrecht; 2016. Available from: https://www.nivel.nl/nl/publicatie/nivel-primary-care-database-sentinel-practices-2015

[15] Smit T Carstens G Han W Bulsink K de Bakker J Elahi M Flexible and scalable participatory syndromic and virological surveillance for respiratory infections: our experiences in The Netherlands. medRxiv. 2024:2024.04.24.24306278 . 10.1101/2024.04.24.24306278 PMC12380313 40857244

[16] Meijer A Beerens A Claas E Hermans M de Jong A Molenkamp R Preparing the outbreak assistance laboratory network in the Netherlands for the detection of the influenza virus A(H1N1) variant. J Clin Virol. 2009;45(3):179-84. 10.1016/j.jcv.2009.06.003 19540155

[17] Mackenzie GA Vilane A Salaudeen R Hogerwerf L van den Brink S Wijsman LA Respiratory syncytial, parainfluenza and influenza virus infection in young children with acute lower respiratory infection in rural Gambia. Sci Rep. 2019;9(1):17965. 10.1038/s41598-019-54059-4 31784567 PMC6884537

[18] van der Vries E Germeraad EA Kroneman A Dieste Pérez L Eggink D Willems E Swine influenza virus surveillance program to assess the risk for animal and public health in the Netherlands, 2022-2023. Euro Surveill. 2025; (Forthcoming). 10.2807/1560-7917.ES.2025.30.22.2400664 PMC12143122 40476289

[19] Zhou B Donnelly ME Scholes DT St George K Hatta M Kawaoka Y Single-reaction genomic amplification accelerates sequencing and vaccine production for classical and Swine origin human influenza a viruses. J Virol. 2009;83(19):10309-13. 10.1128/JVI.01109-09 19605485 PMC2748056

[20] Jonges M van der Lubben IM Dijkstra F Verhoef L Koopmans M Meijer A . Dynamics of antiviral-resistant influenza viruses in the Netherlands, 2005-2008. Antiviral Res. 2009;83(3):290-7. 10.1016/j.antiviral.2009.07.003 19591877

[21] de Jong JC Smith DJ Lapedes AS Donatelli I Campitelli L Barigazzi G Antigenic and genetic evolution of swine influenza A (H3N2) viruses in Europe. J Virol. 2007;81(8):4315-22. 10.1128/JVI.02458-06 17287258 PMC1866135

[22] Myers KP Olsen CW Gray GC . Cases of swine influenza in humans: a review of the literature. Clin Infect Dis. 2007;44(8):1084-8. 10.1086/512813 17366454 PMC1973337

[23] Olsen CW Brammer L Easterday BC Arden N Belay E Baker I Serologic evidence of H1 swine Influenza virus infection in swine farm residents and employees. Emerg Infect Dis. 2002;8(8):814-9. 10.3201/eid0808.010474 12141967 PMC2732505

[24] European Centre for Disease Prevention and Control (ECDC). Zoonotic influenza - Annual Epidemiological Report for 2022. Stockholm: ECDC; 23 May 2023. Available from: https://www.ecdc.europa.eu/en/swine-influenza/surveillance-and-disease-data

[25] Centers for Disease Control and Prevention (CDC). Confirmed Novel Influenza A Virus Infections. Atlanta: CDC. [Accessed: 14 May 2025]. Available from: https://gis.cdc.gov/grasp/fluview/Novel_Influenza.html

[26] World Health Organization (WHO). 2023: outbreaks of swine influenza. Geneva: WHO; 30 Mar 2024. Available from: https://www.who.int/news/item/30-03-2024-2023--outbreaks-of-swine-influenza

[27] Meng F Chen Y Song Z Zhong Q Zhang Y Qiao C Continued evolution of the Eurasian avian-like H1N1 swine influenza viruses in China. Sci China Life Sci. 2023;66(2):269-82. 10.1007/s11427-022-2208-0 36219302

[28] Teirlinck AC de Gier B Meijer A Donker G de Lange M Koppeschaar C The incidence of symptomatic infection with influenza virus in the Netherlands 2011/2012 through 2016/2017, estimated using Bayesian evidence synthesis. Epidemiol Infect. 2018;147:e30. 10.1017/S095026881800273X 30348244 PMC6518592

[29] World Health Organization (WHO). Laboratory methodologies for testing the antiviral susceptibility of influenza viruses: Neuraminidase inhibitor (NAI). Geneva: WHO. [Accessed: 14 May 2025]. Available from: https://www.who.int/teams/global-influenza-programme/laboratory-network/quality-assurance/antiviral-susceptibility-influenza/neuraminidase-inhibitor

