# Author’s correction for Euro Surveill. 2025;30(19)

**DOI:** 10.2807/1560-7917.ES.2025.30.43.251030c

**Published:** 2025-10-30

**Authors:** Eurosurveillance editorial team

**Affiliations:** 1 European Centre for Disease Prevention and Control (ECDC), Stockholm, Sweden

In the article ‘Human infections with Eurasian avian-like swine influenza virus detected by coincidence via routine respiratory surveillance systems, the Netherlands, 2020 to 2023’ by Eggink et al., published on 15 May 2025, the name of Mariëtte Hooiveld was incorrectly spelled at the time of publication. This has been corrected on 28 October 2025 at the request of the authors.

