# Supplementary material for: Human infections with Eurasian avian-like swine influenza virus detected by coincidence via routine respiratory surveillance systems, the Netherlands, 2020 to 2023
Source: Euro Surveill. 2025 May 15;30(19):2400662. doi: 10.2807/1560-7917.ES.2025.30.19.2400662 (PMC12083066; doi:10.2807/1560-7917.ES.2025.30.19.2400662)
Supplement: Supplementary Material [file 24-00662_EGGINK_Supplement.pdf]

"This **supplementary material** is hosted by *Eurosurveillance* as supporting information alongside the article *“Human infections with Eurasian Avian-like swine influenza virus in The Netherlands detected by coincidence via routine respiratory surveillance systems, 2020-2023”*, on behalf of the authors, who remain responsible for the accuracy and appropriateness of the content. The same standards for ethics, copyright, attributions and permissions as for the article apply. Supplements are not edited by *Eurosurveillance* and the journal is not responsible for the maintenance of any links or email addresses provided therein."

Title: Human infections with Eurasian Avian-like swine influenza virus in The Netherlands detected by coincidence via routine respiratory surveillance systems, 2020-2023

Authors: Dirk Eggink[1], Annelies Kroneman[1], Jozef Dingemans[2], Gabriel Goderski[1], Sharon van den Brink[1], Mariam Bagheri[1], Pascal Lexmond[3], Mark Pronk[3], Erhard van der Vries[5], Evelien Germeraad[4], Diederik Brandwagt[1], Manon Houben[5], Mariëtte van Hooiveld[6], Joke van der Giessen[1], Rianne van Gageldonk-Lafeber[1], Ron Fouchier[3], Adam Meijer[1]

Affiliations:
1. Centre for Infectious Disease Control, National Institute for Public Health and Environment (RIVM), Bilthoven, the Netherlands
2. Department of Medical Microbiology, Infectious Diseases & Infection Prevention, Care and Public Health Research Institute (CAPHRI), Maastricht University Medical Center, Maastricht, The Netherland
3. Department of Viroscience, Erasmus Medical Center, Rotterdam, The Netherlands
4. Wageningen Bioveterinary Research (WBVR), Lelystad, The Netherlands
5. Royal GD, Deventer, The Netherlands
6. Nivel, Utrecht, The Netherlands

Figure S1A Phylogenetic tree swIAV PB2.
Human cases of swIAV infection from The Netherlands are depicted in blue, with the three cases described indicated by their strain name. Sequences from the recently established One Health influenza surveillance system (19) are depicted in orange and human seasonal vaccine strains, both H1N1 prior to 2009 and A(H1N1)pdm09, are indicated in pink. Sequences originating from the swAIV isolated from pigs related to the likely source of case 2 care depicted in green.

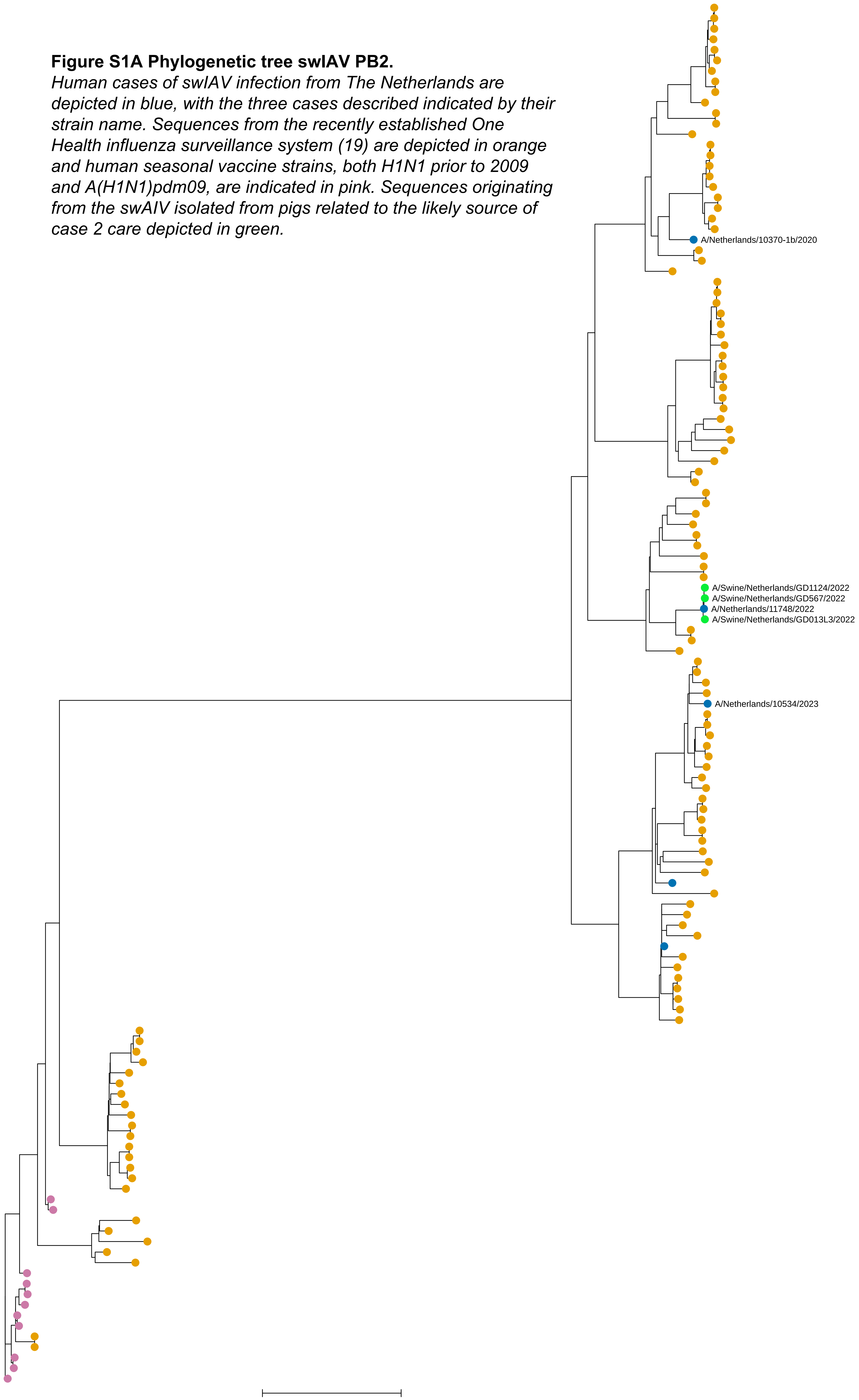

Figure S1B Phylogenetic tree swIAV PB2 - containing all complete strain names.
Human cases of swIAV infection from The Netherlands are depicted in blue, with the three cases described indicated by their strain name. Sequences from the recently established One Health influenza surveillance system (19) are depicted in orange and human seasonal vaccine strains, both H1N1 prior to 2009 and A(H1N1)pdm09, are indicated in pink. Sequences originating from the swAIV isolated from pigs related to the likely source of case 2 care depicted in green.

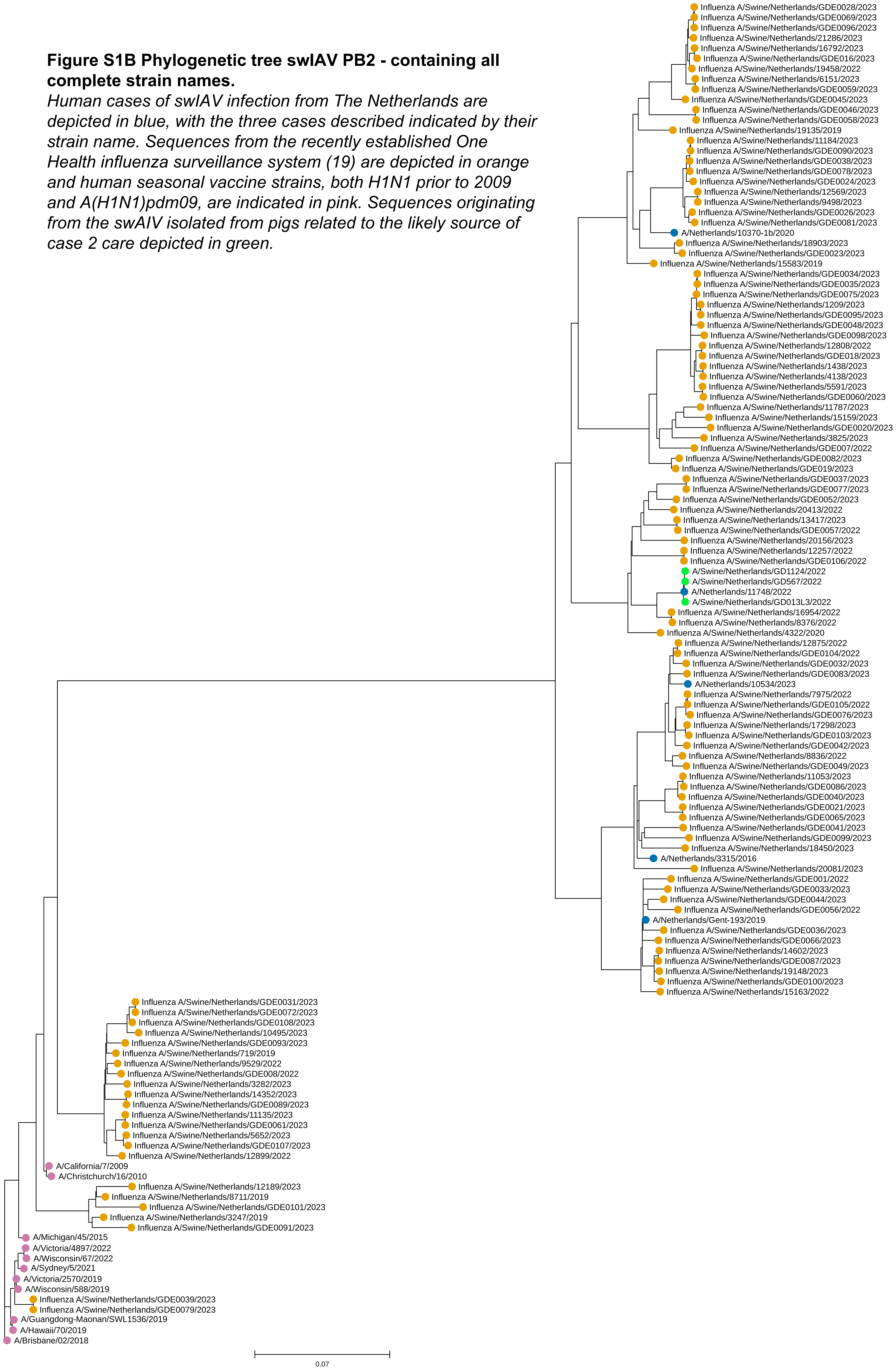

Figure S2 Phylogenetic tree swIAV PB1.

Human cases of swIAV infection from The Netherlands are depicted in blue, with the three cases described indicated by their strain name. Sequences from the recently established One Health influenza surveillance system (19) are depicted in orange and human seasonal vaccine strains, both H1N1 prior to 2009 and A(H1N1)pdm09, are indicated in pink. Sequences originating from the swAIV isolated from pigs related to the likely source of case 2 care depicted in green.

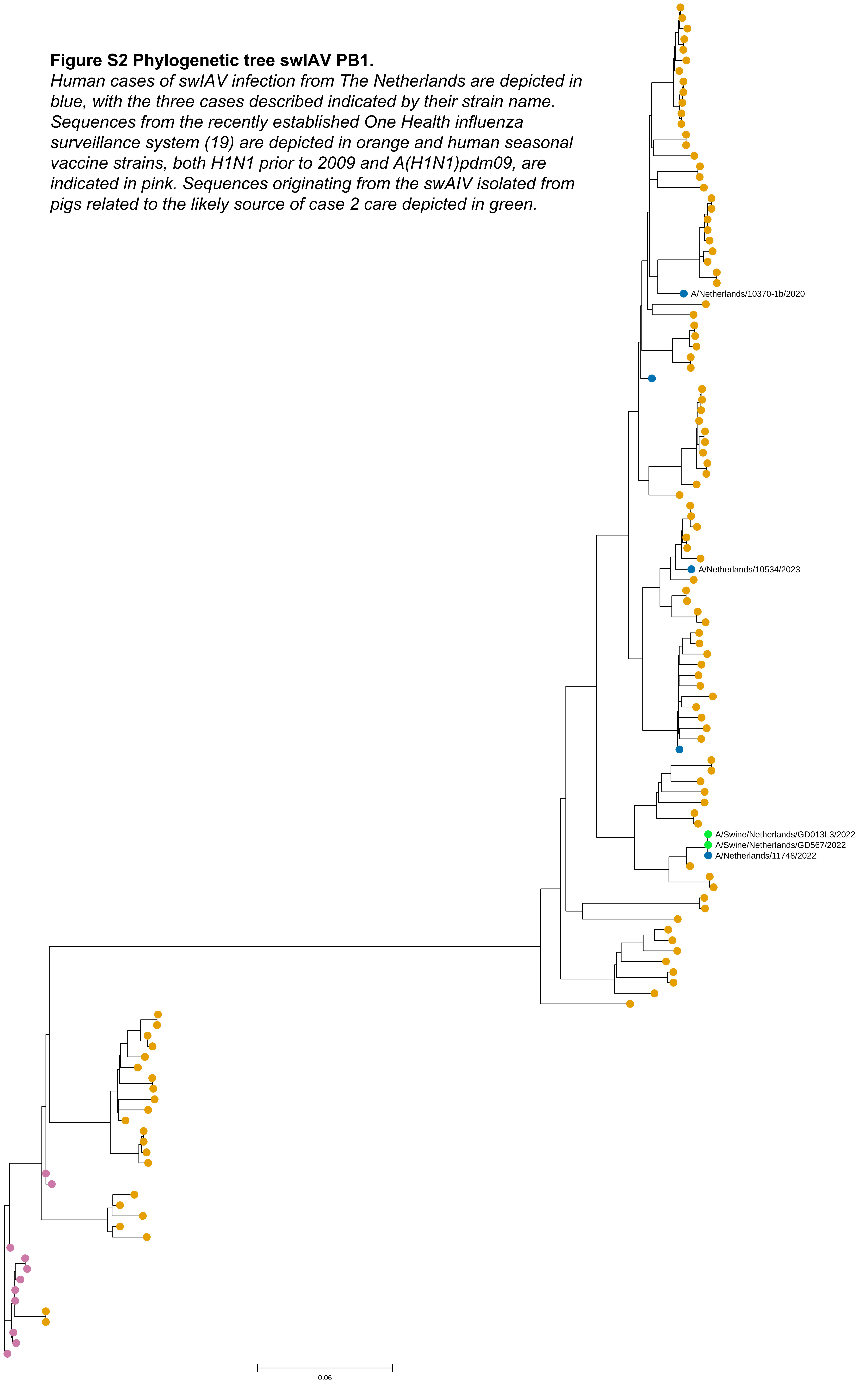

Figure S2B Phylogenetic tree swIAV PB1 - containing all complete strain names.

Human cases of swIAV infection from The Netherlands are depicted in blue, with the three cases described indicated by their strain name. Sequences from the recently established One Health influenza surveillance system (19) are depicted in orange and human seasonal vaccine strains, both H1N1 prior to 2009 and A(H1N1)pdm09, are indicated in pink. Sequences originating from the swAIV isolated from pigs related to the likely source of case 2 care depicted in green.

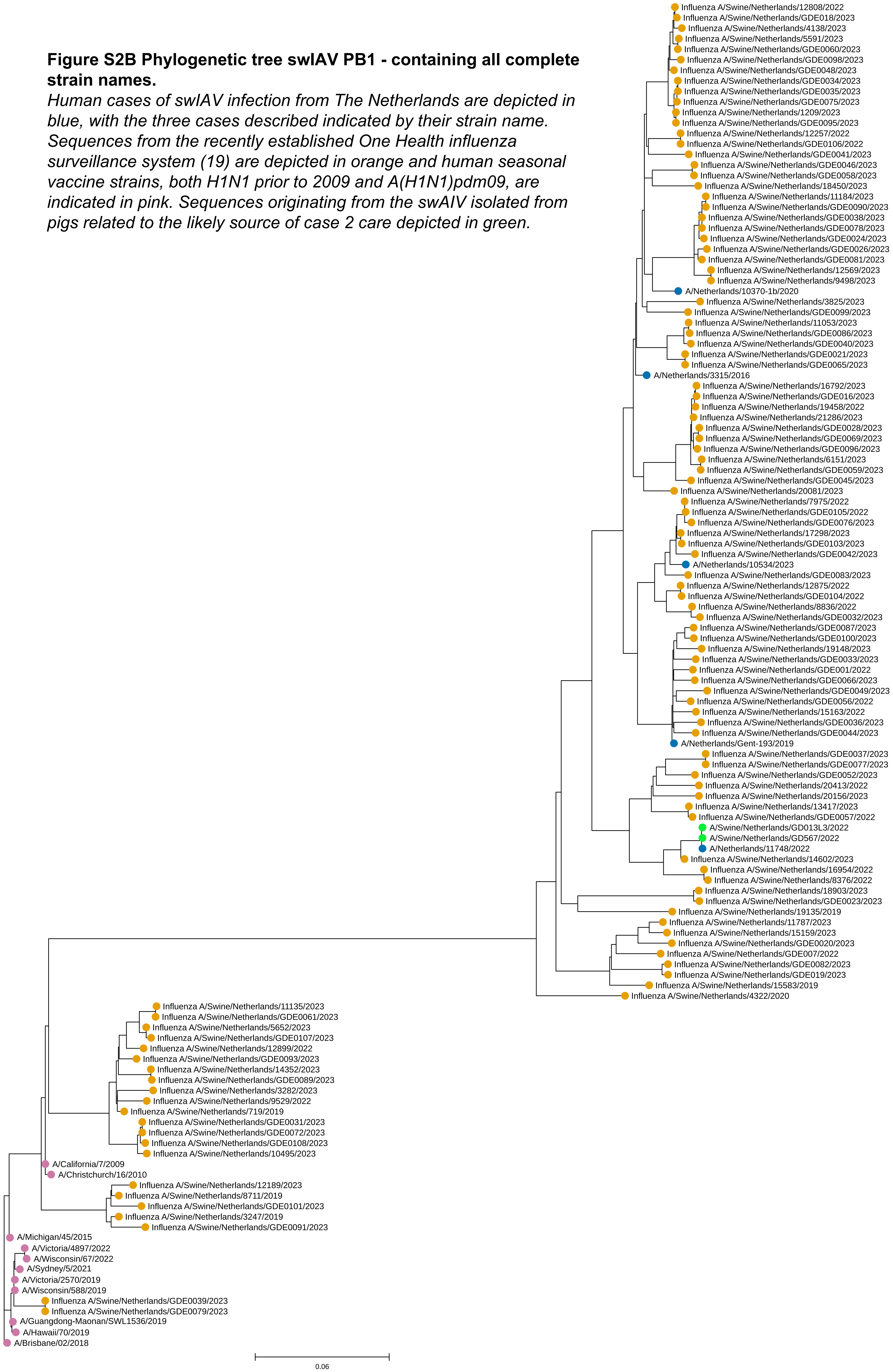

Figure S3A Phylogenetic tree swIAV PA.
Human cases of swIAV infection from The Netherlands are depicted in blue, with the three cases described indicated by their strain name. Sequences from the recently established One Health influenza surveillance system (19) are depicted in orange and human seasonal vaccine strains, both H1N1 prior to 2009 and A(H1N1)pdm09, are indicated in pink. Sequences originating from the swAIV isolated from pigs related to the likely source of case 2 care depicted in green.

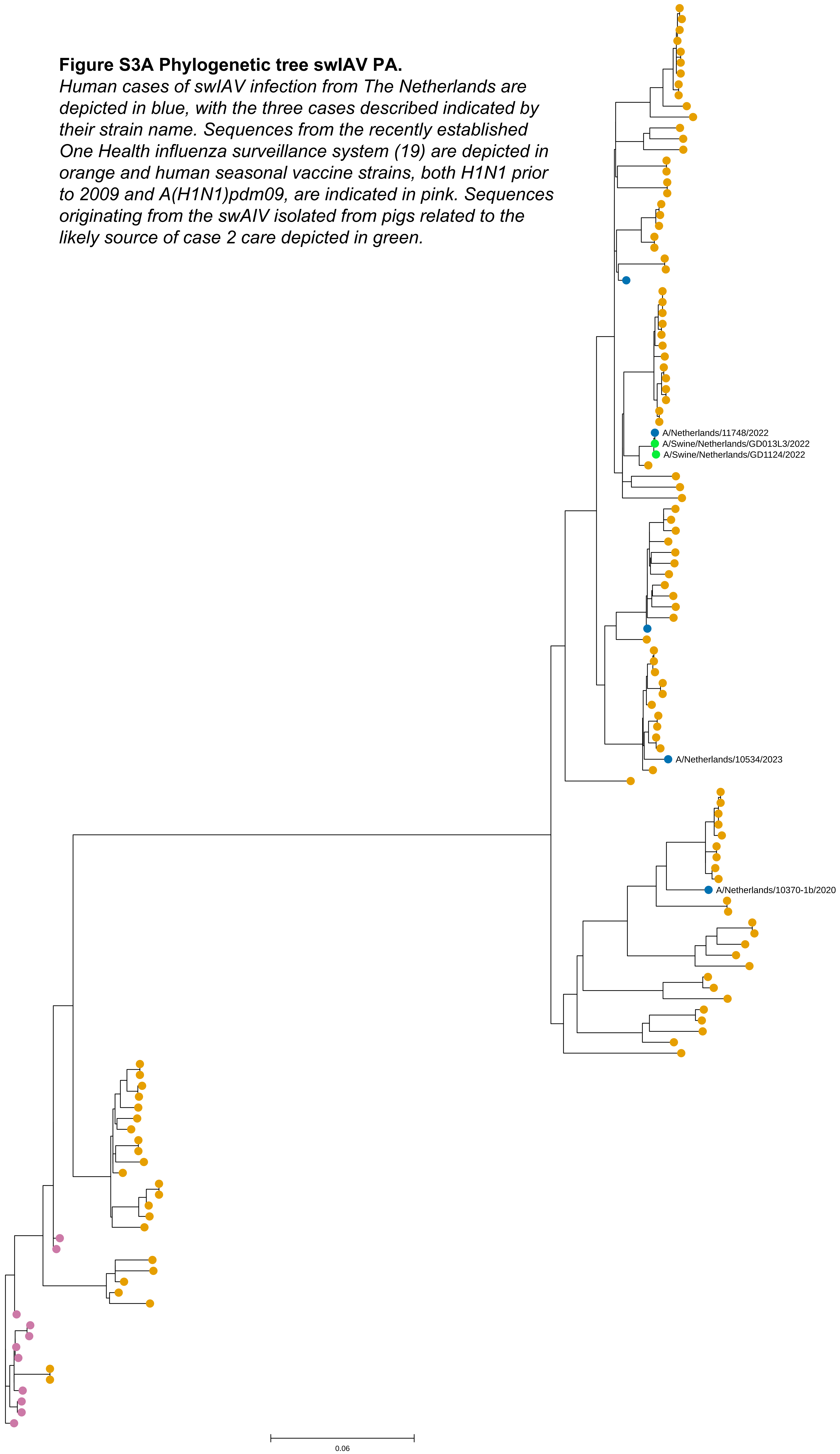

Figure S3B Phylogenetic tree swIAV PA - containing all complete strain names.

Human cases of swIAV infection from The Netherlands are depicted in blue, with the three cases described indicated by their strain name. Sequences from the recently established One Health influenza surveillance system (19) are depicted in orange and human seasonal vaccine strains, both H1N1 prior to 2009 and A(H1N1)pdm09, are indicated in pink. Sequences originating from the swAIV isolated from pigs related to the likely source of case 2 care depicted in green.

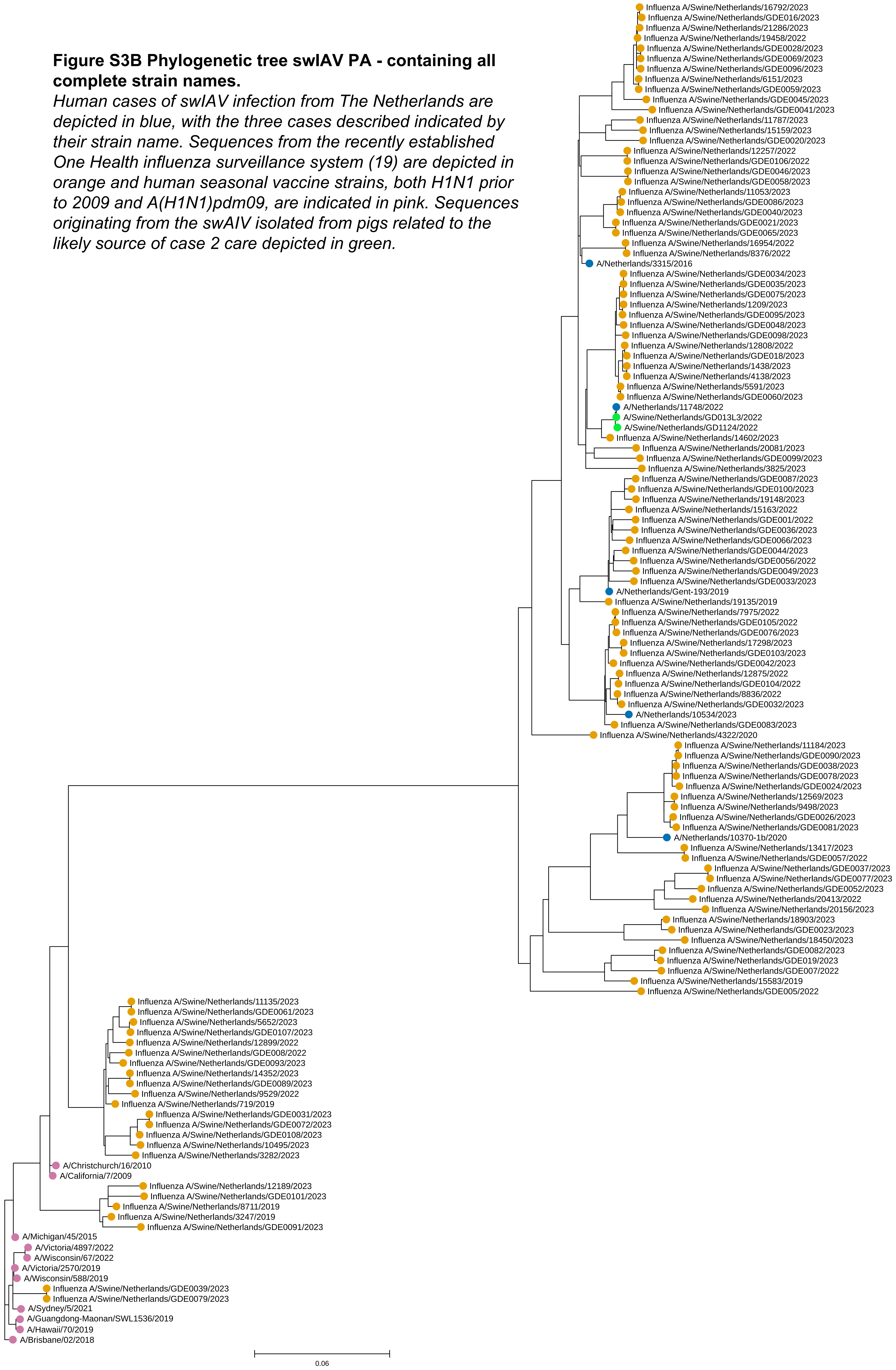

Figure S4A Phylogenetic tree swIAV NP.

Human cases of swIAV infection from The Netherlands are depicted in blue, with the three cases described indicated by their strain name. Sequences from the recently established One Health influenza surveillance system (19) are depicted in orange and human seasonal vaccine strains, both H1N1 prior to 2009 and A(H1N1)pdm09, are indicated in pink. Sequences originating from the swAIV isolated from pigs related to the likely source of case 2 care depicted in green.

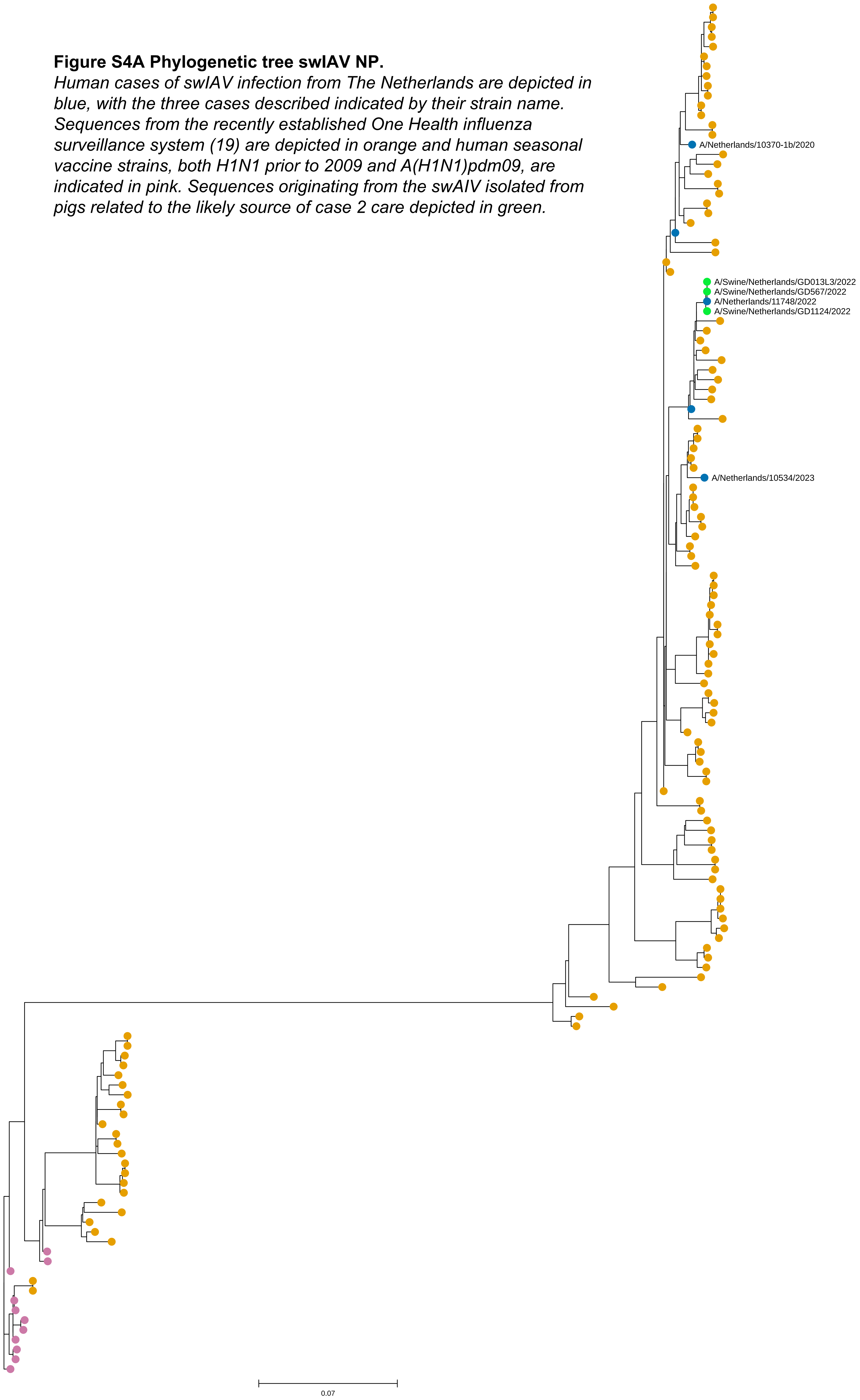

Figure S4B Phylogenetic tree swIAV NP - containing all complete strain names.

Human cases of swIAV infection from The Netherlands are depicted in blue, with the three cases described indicated by their strain name. Sequences from the recently established One Health influenza surveillance system (19) are depicted in orange and human seasonal vaccine strains, both H1N1 prior to 2009 and A(H1N1)pdm09, are indicated in pink. Sequences originating from the swAIV isolated from pigs related to the likely source of case 2 care depicted in green.

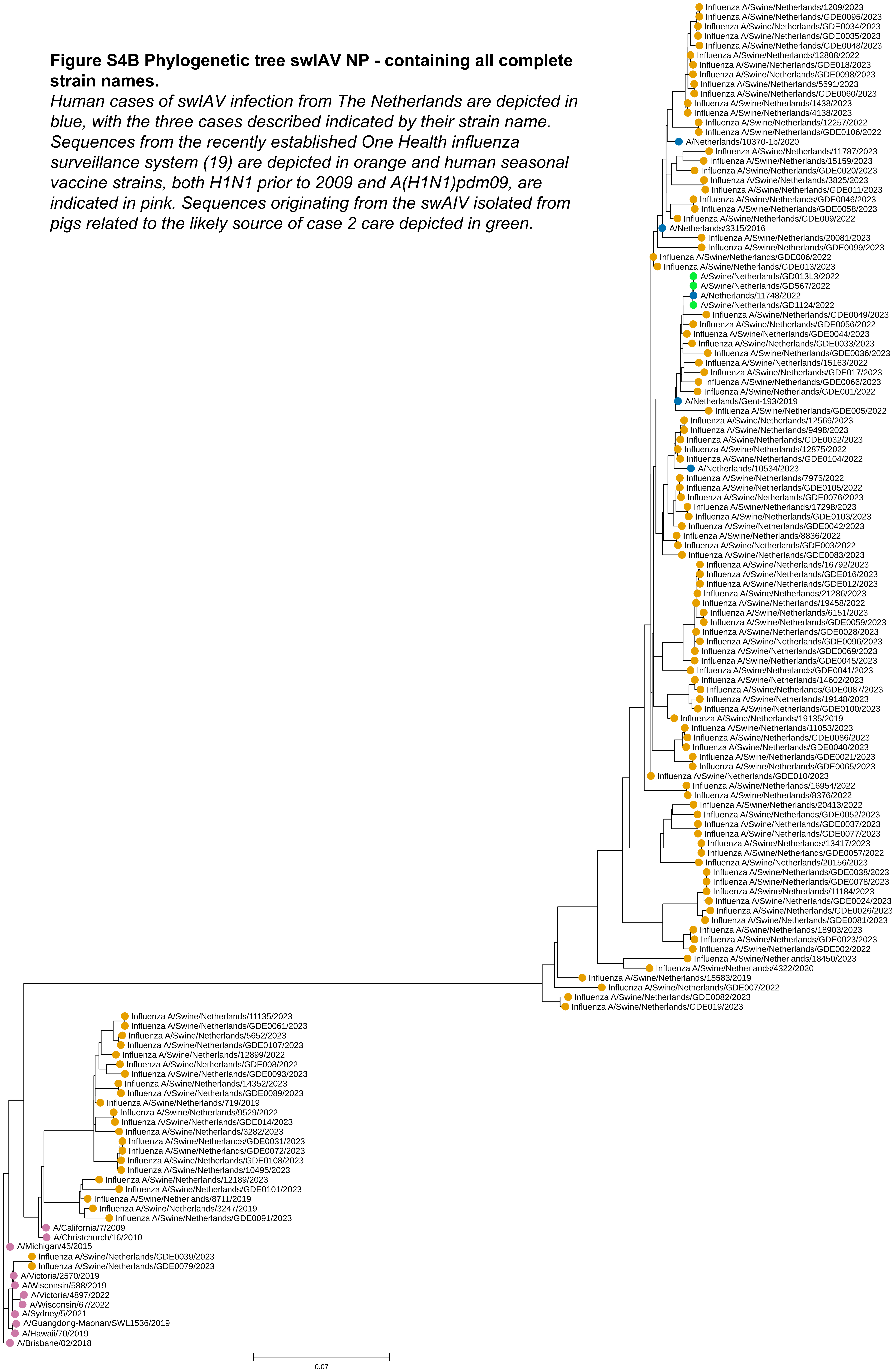

Figure S5A Phylogenetic tree swIAV NA (N1).
Human cases of swIAV infection from The Netherlands are depicted in blue, with the three cases described indicated by their strain name. Sequences from the recently established One Health influenza surveillance system (19) are depicted in orange and human seasonal vaccine strains, both H1N1 prior to 2009 and A(H1N1)pdm09, are indicated in pink. Sequences originating from the swAIV isolated from pigs related to the likely source of case 2 care depicted in green.

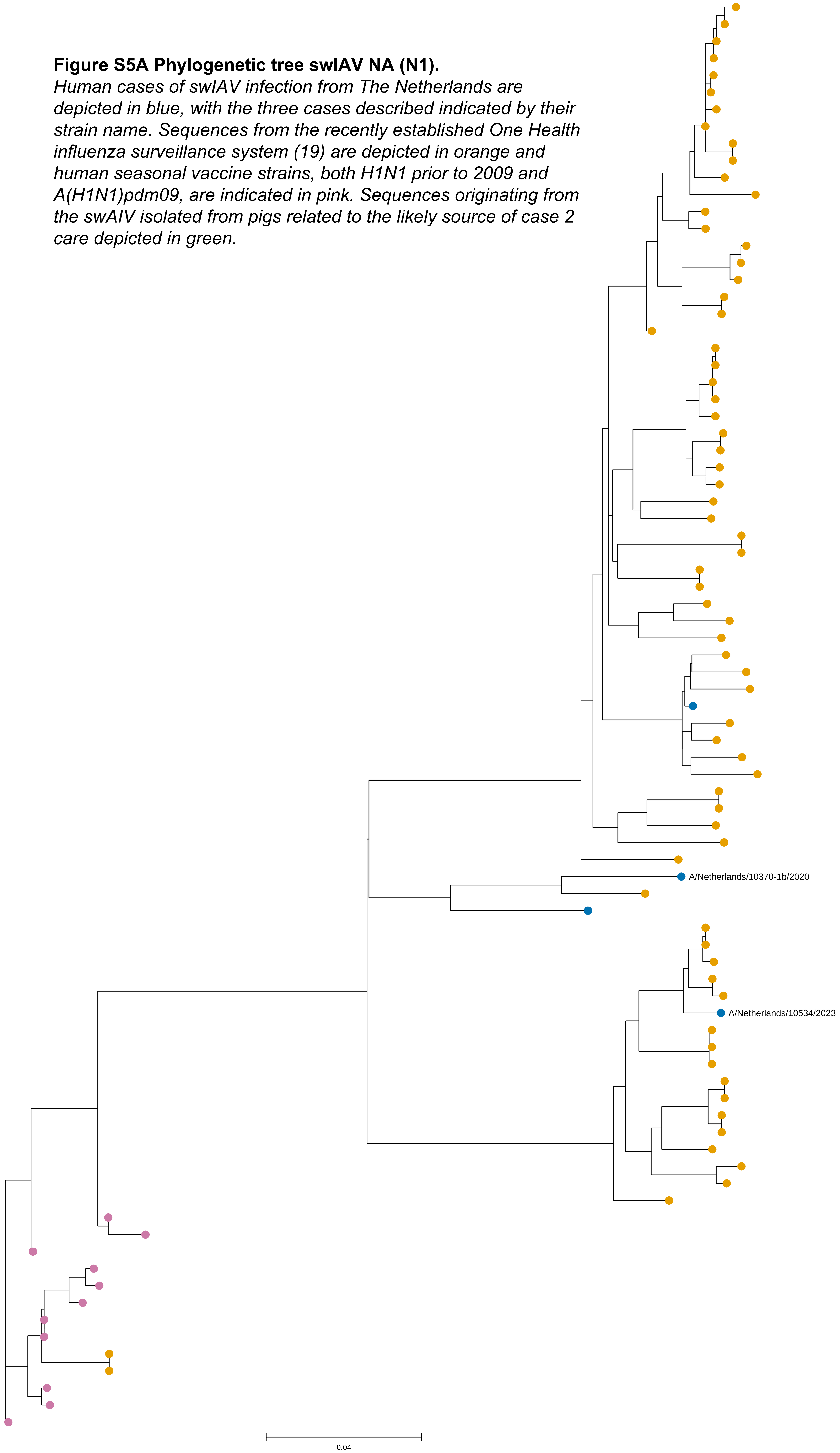

Figure S5B Phylogenetic tree swIAV NA (N1) - containing all complete strain names.

Human cases of swIAV infection from The Netherlands are depicted in blue, with the three cases described indicated by their strain name. Sequences from the recently established One Health influenza surveillance system (19) are depicted in orange and human seasonal vaccine strains, both H1N1 prior to 2009 and A(H1N1)pdm09, are indicated in pink. Sequences originating from the swAIV isolated from pigs related to the likely source of case 2 care depicted in green.

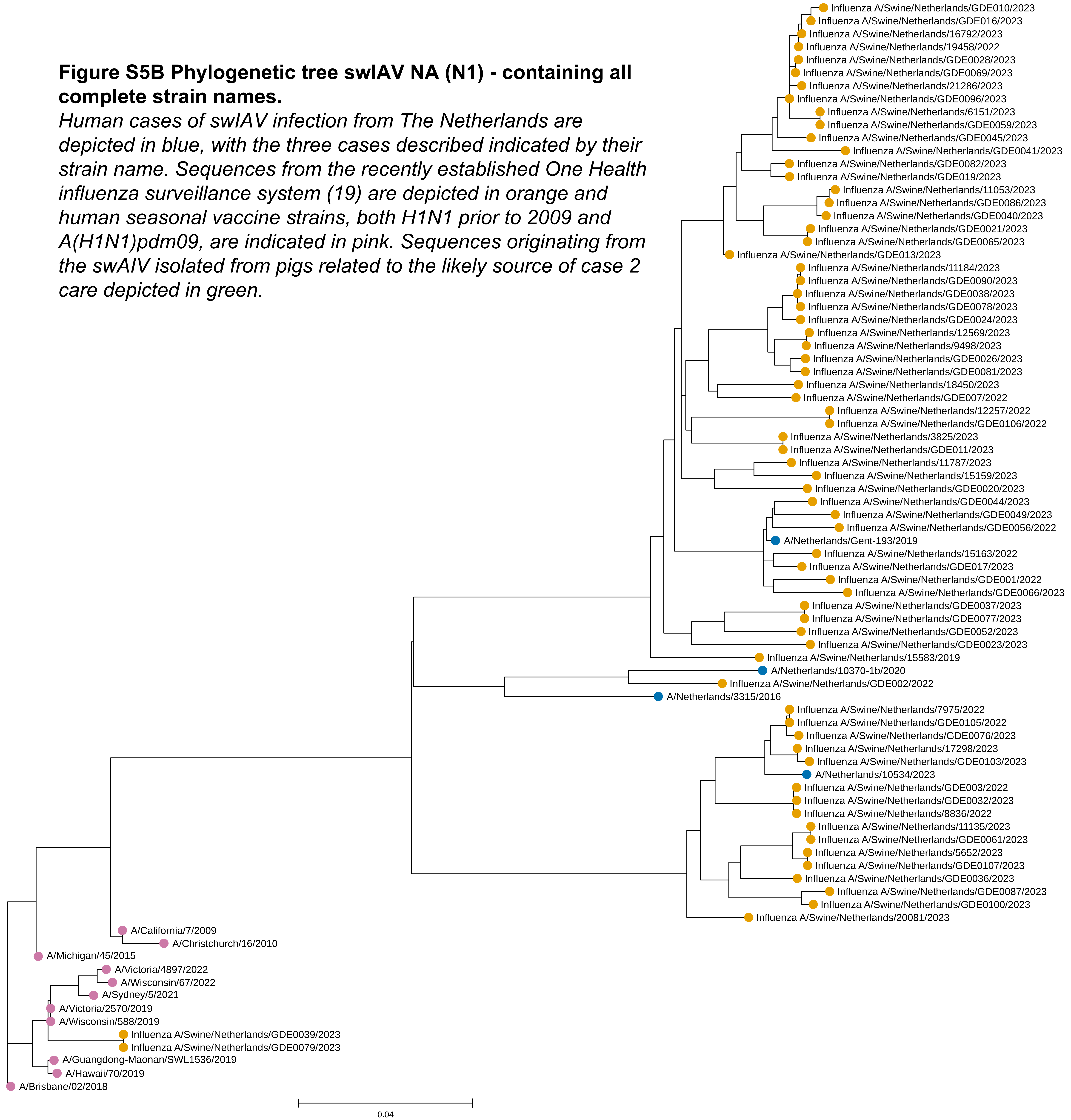

Figure S6A Phylogenetic tree swIAV NA (N2).
Human cases of swIAV infection from The Netherlands are depicted in blue, with the three cases described indicated by their strain name. Sequences from the recently established One Health influenza surveillance system (19) are depicted in orange and human seasonal vaccine strains, both H1N1 prior to 2009 and A(H1N1)pdm09, are indicated in pink. Sequences originating from the swAIV isolated from pigs related to the likely source of case 2 care depicted in green.

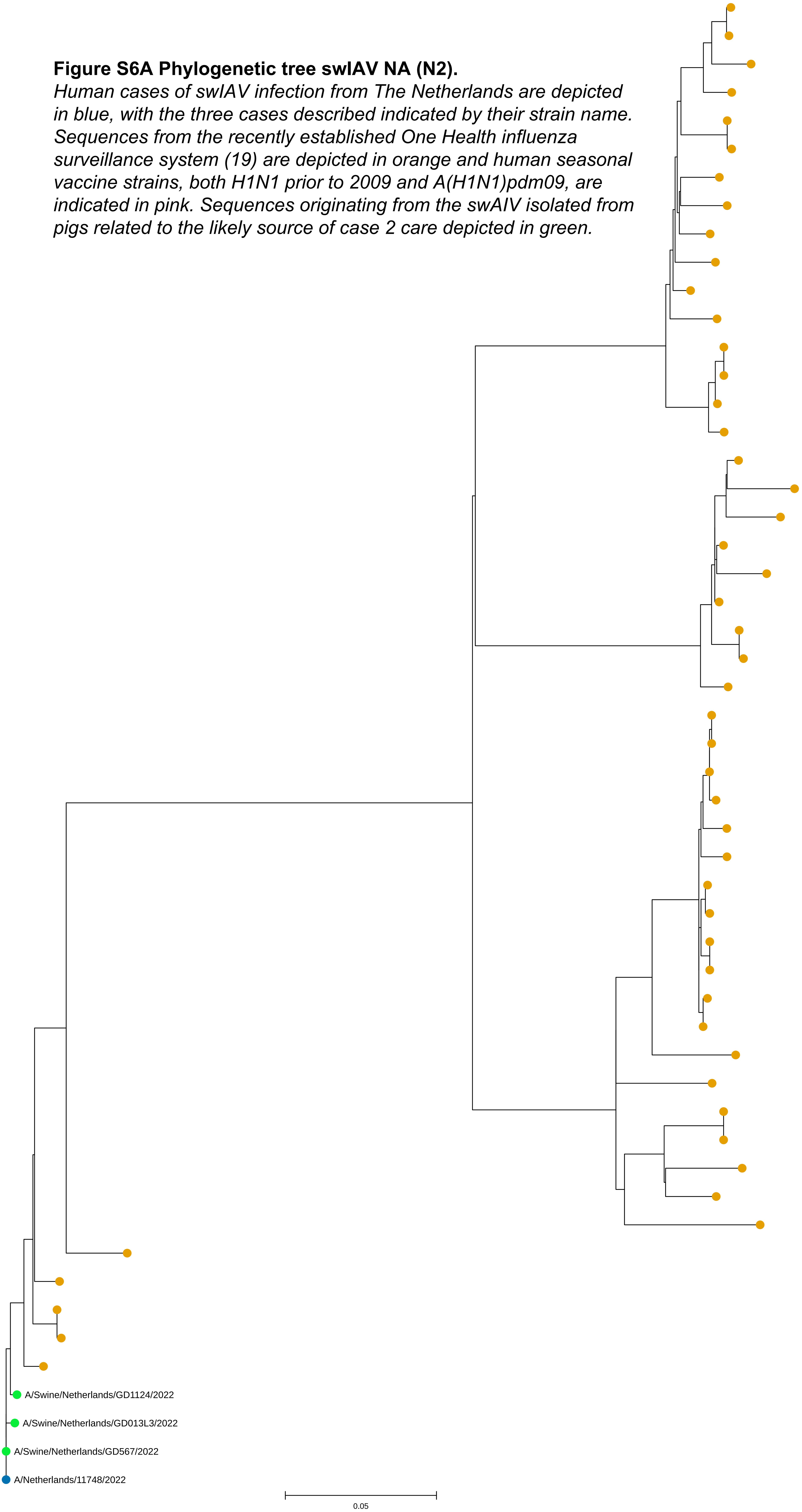

Figure S6B Phylogenetic tree swIAV NA (N2) - containing all complete strain names.
Human cases of swIAV infection from The Netherlands are depicted in blue, with the three cases described indicated by their strain name. Sequences from the recently established One Health influenza surveillance system (19) are depicted in orange and human seasonal vaccine strains, both H1N1 prior to 2009 and A(H1N1)pdm09, are indicated in pink. Sequences originating from the swAIV isolated from pigs related to the likely source of case 2 care depicted in green.

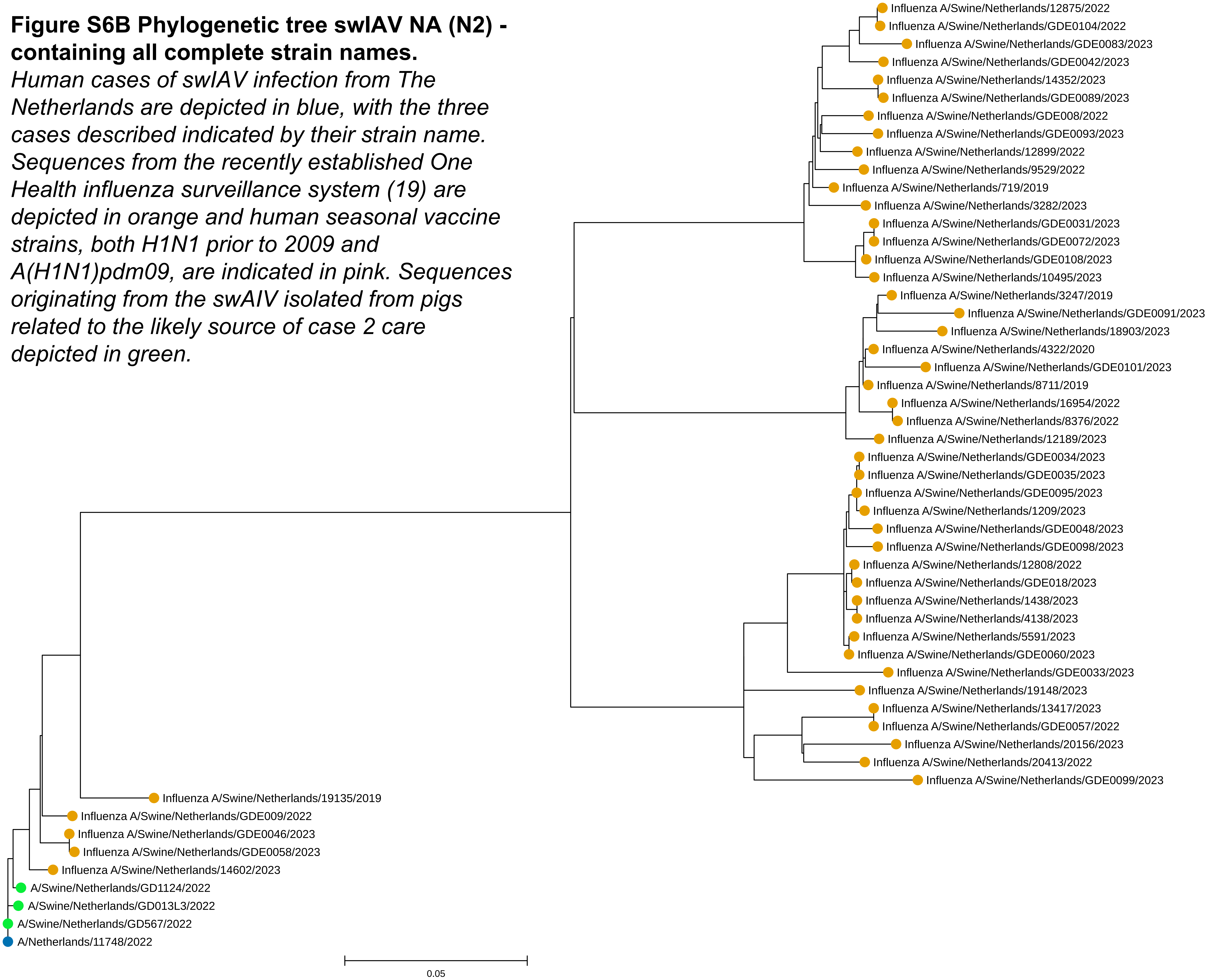

Figure S7A Phylogenetic tree swIAV M.

Human cases of swIAV infection from The Netherlands are depicted in blue, with the three cases described indicated by their strain name. Sequences from the recently established One Health influenza surveillance system (19) are depicted in orange and human seasonal vaccine strains, both H1N1 prior to 2009 and A(H1N1)pdm09, are indicated in pink. Sequences originating from the swAIV isolated from pigs related to the likely source of case 2 care depicted in green.

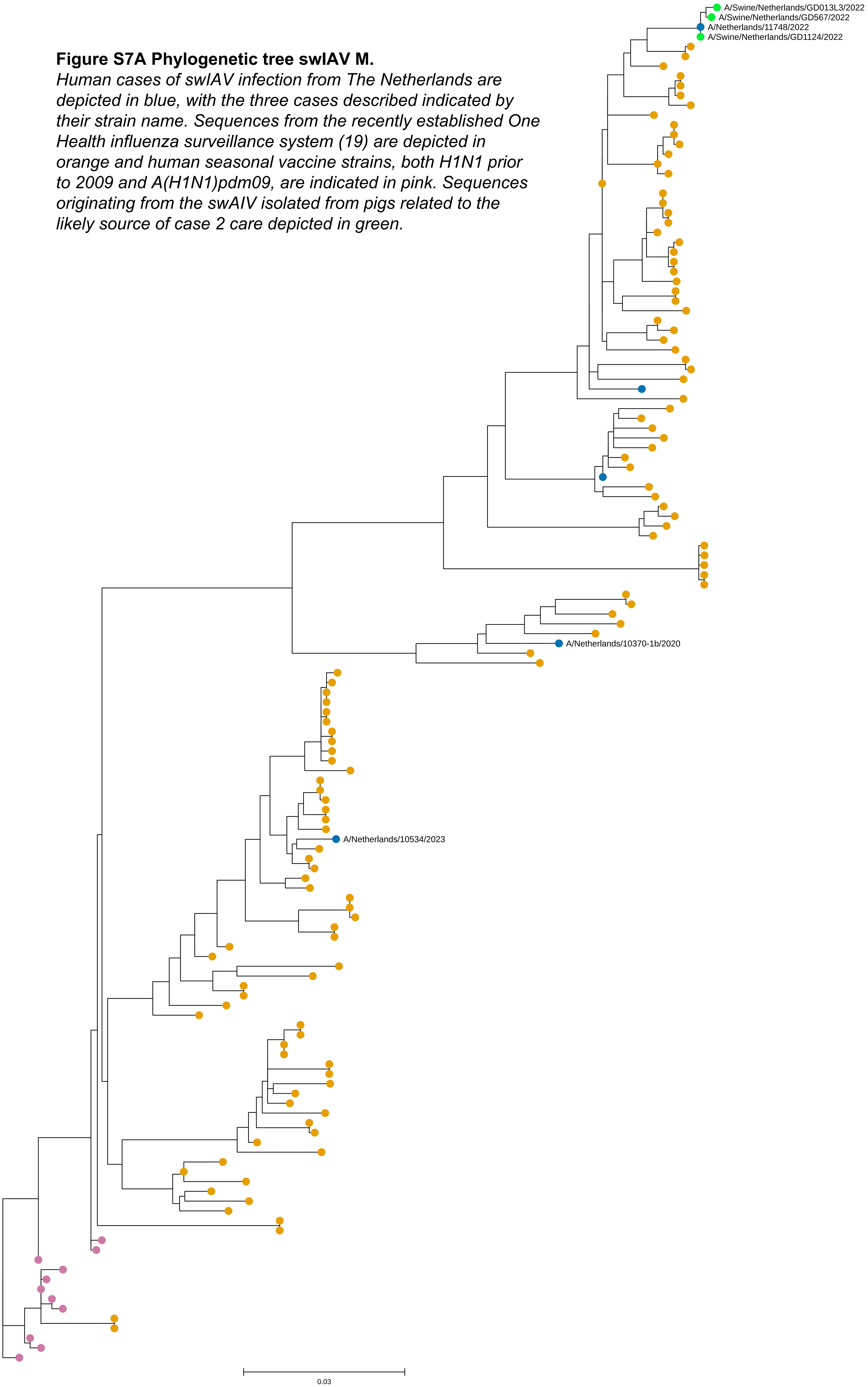

Figure S7B Phylogenetic tree swIAV M - containing all complete strain names.

Human cases of swIAV infection from The Netherlands are depicted in blue, with the three cases described indicated by their strain name. Sequences from the recently established One Health influenza surveillance system (19) are depicted in orange and human seasonal vaccine strains, both H1N1 prior to 2009 and A(H1N1)pdm09, are indicated in pink. Sequences originating from the swAIV isolated from pigs related to the likely source of case 2 care depicted in green.

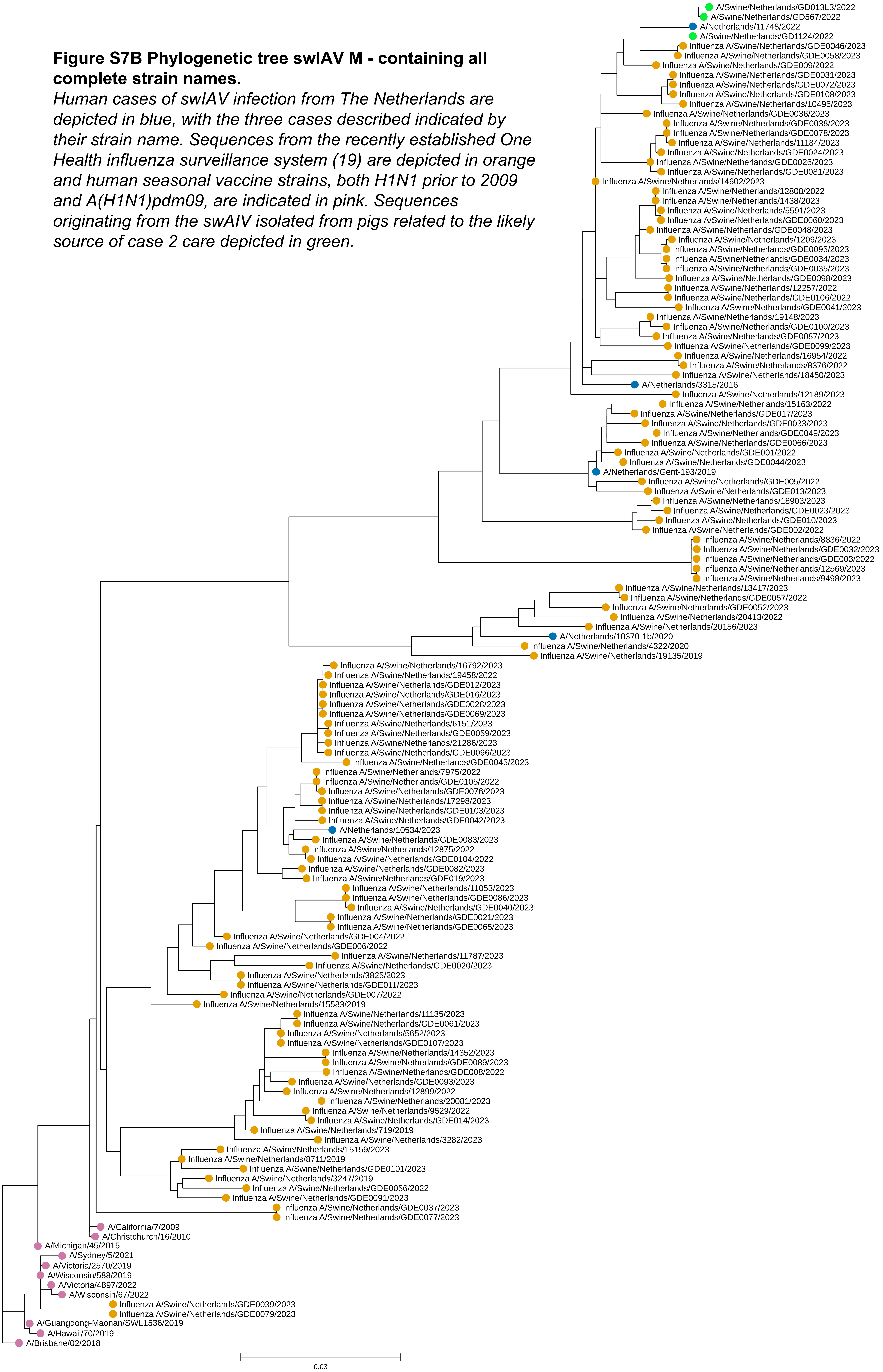

Figure S8A Phylogenetic tree swIAV NS.
Human cases of swIAV infection from The Netherlands are depicted in blue, with the three cases described indicated by their strain name. Sequences from the recently established One Health influenza surveillance system (19) are depicted in orange and human seasonal vaccine strains, both H1N1 prior to 2009 and A(H1N1)pdm09, are indicated in pink. Sequences originating from the swAIV isolated from pigs related to the likely source of case 2 care depicted in green.

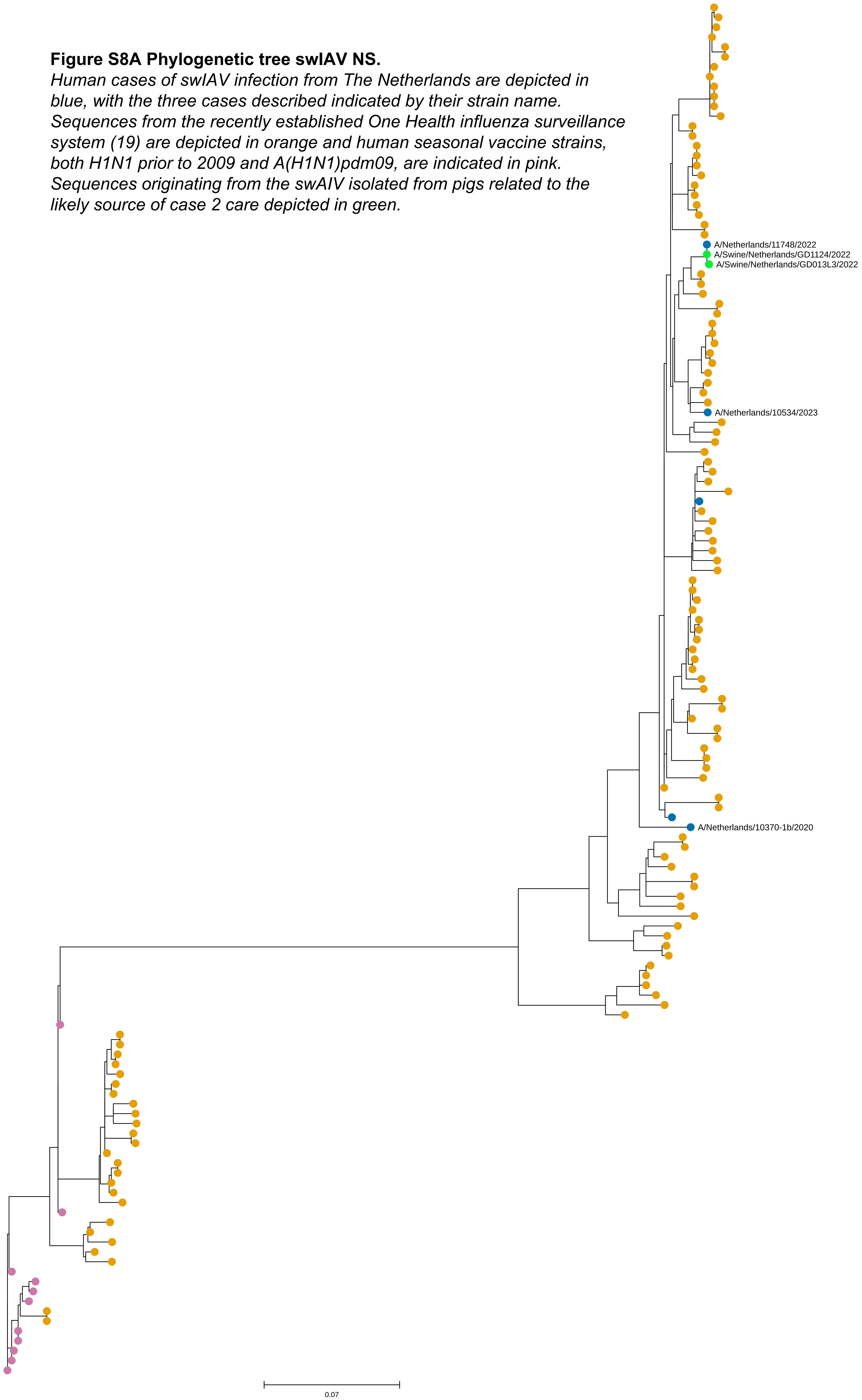

Figure S8B Phylogenetic tree swIAV NS - containing all complete strain names.

Human cases of swIAV infection from The Netherlands are depicted in blue, with the three cases described indicated by their strain name. Sequences from the recently established One Health influenza surveillance system (19) are depicted in orange and human seasonal vaccine strains, both H1N1 prior to 2009 and A(H1N1)pdm09, are indicated in pink. Sequences originating from the swAIV isolated from pigs related to the likely source of case 2 care depicted in green.

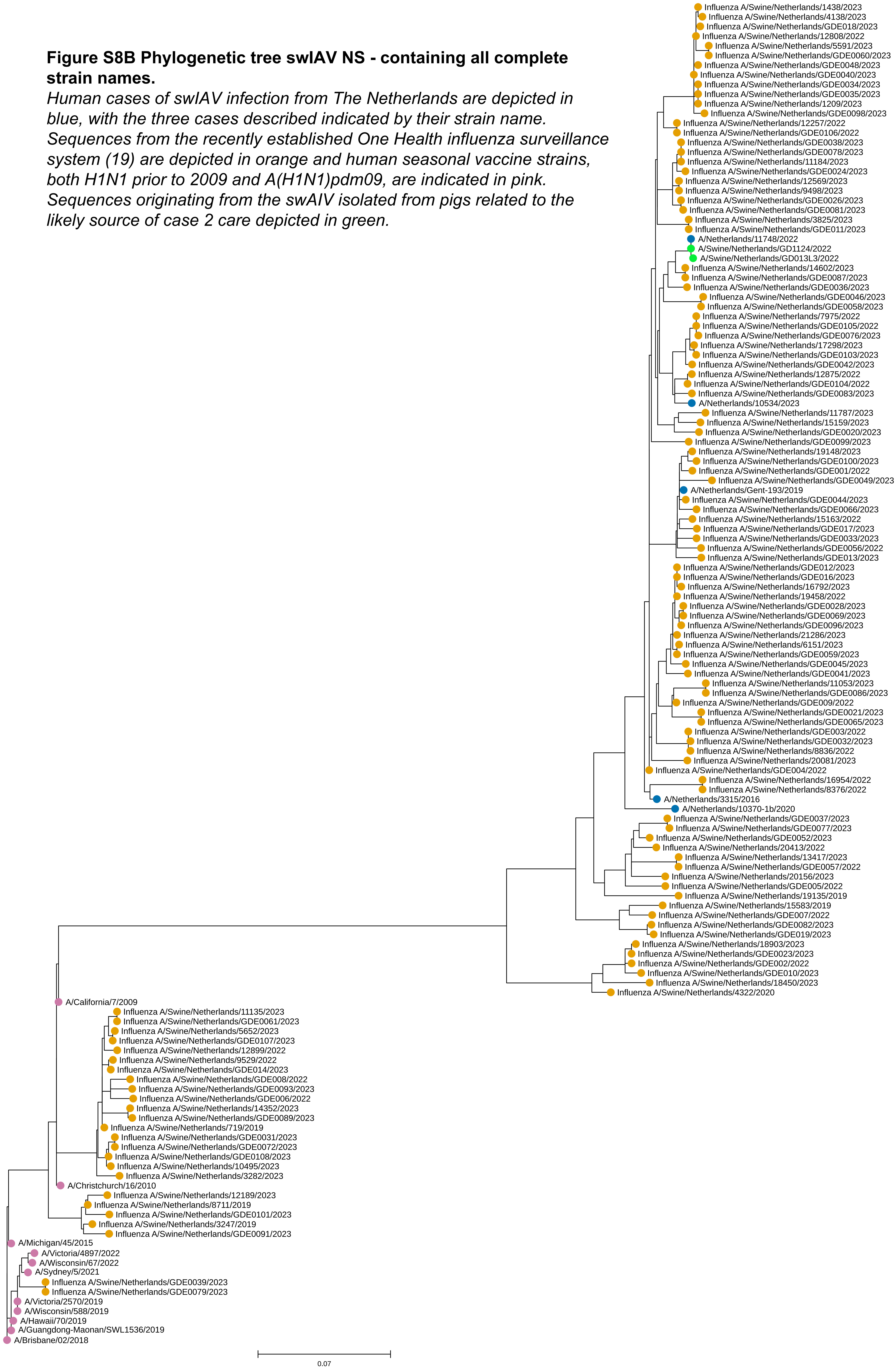

Table S1.

Antigenic characterization of isolates from human cases with Eurasian Avian-like swine influenza virus from the Netherlands by HI assay with turkey erythrocytes and ferret antisera. Homologous titers are shown in bold and underlined. H1 classical swine lineage (clade 1A), including H1pdm09-like viruses are depicted in green, H1 human seasonal lineage (clade 1B) are depicted in blue and H1 Eurasian avian-like lineage (clade 1C), including the three human cases described are depicted in orange.

			Sw/NL/5591/23	Sw/NL/8836/22	Sw/NL/4322/20	Sw/NL/719/19	Cal/7/09	Sw/Best/5M/96	Hebei/1572/19	Sw/NL/11135/23	Sw/NL/6151/23	Hessen/47/20	NL/386/86	NL/3315/16	NL/10370-1b/20
			FS23002	FS23004	FS23007	FS23005	FS10003	F571		FS23011	FS23009	F02/21	F562/3	F21/17	F18/21
Sw/NL/5591/23	H1N2	1B.1.2.1	**2560**	160	<10	<10	160	<10	<10	<10	<10	<10	120	<10	<10
Sw/NL/8836/22	H1N1	1B.1.2.1	320	**3840**	<10	<10	<10	<10	<10	<10	<10	<10	<10	<10	<10
Sw/NL/4322/20	H1N2	1A.3.3.2	<10	<10	**640**	<10	<10	<10	<10	<10	<10	<10	<10	<10	<10
Sw/NL/719/19	H1N2	1A.3.3.2	<10	<10	<10	**2560**	960	<10	320	80	240	640	80	<10	160
Cal/7/09	H1N1pdm	1A.3.3.2	<10	<10	<10	2560	**7680**	<10	1920	480	960	1920	80	<10	960
A/swine/Best/5M/1996	H1N1	1C.2	NT	NT	NT	NT	<10	**1920**	320	NT	NT	10	1280	240	40
Hebei-haigang/1572/19	H1N1v	1C.2.3	<10	<10	<10	1920	2560	<10	**2560**	480	960	2560	240	240	960
Sw/NL/11135/23	H1N1	1C.2.1	<10	40	<10	480	2560	<10	1920	**1280**	480	960	160	240	960
Sw/NL/6151/23	H1N1	1C.2.2	<10	<10	<10	320	1280	<10	640	240	**1920**	640	80	40	480
Hessen/47/20	H1N1v	1C.2.2	<10	<10	<10	960	960	<10	2560	120	480	**3840**	80	<10	320
NL/386/1986	H1N1v	1C	<10	<10	<10	960	1920	240	1920	320	1280	1920	**480**	20	1280
NL/477/1993	H1N1v	1C	<10	<10	<10	480	1920	NT	640	240	640	640	NT	20	640
NL/3315/16	H1N1v	1C.2.1	<10	<10	<10	<10	<10	40	640	480	480	40	240	**2560**	320
NL/10370-1b/20	H1N1v	1C.2.1	<10	<10	<10	320	960	<10	1920	640	640	960	80	320	**1280**
NL/10534/23	H1N1v	1C.2.2	<10	<10	<10	640	1280	NT	1280	320	1280	1280	NT	40	640
NL/11748/22	H1N2v	1C.2.2	<10	<10	<10	1920	1920	NT	1920	480	1920	2560	NT	640	320
NL/Gent-193/19	H1N1v	1C.2.2	<10	<10	<10	1280	1920	NT	2560	240	1280	2560	NT	640	320

Table S2.

Primer sequences used in diagnostic PCR, subtyping PCR and nanopore sequencing.

Detectie PCR

Influenza A (RIVM)	Sequence	Label
INFAM-sense	AAG ACC AAT C**C**T GTC ACC TCT GA	
INFAM-sense3	AAG ACC AAT C**T**T GTC ACC TCT GA	
INFAM-sense4.1	AAG ACC AAT **T**CT GTC ACC T**Y**T GA	
INFAM A-sense	**C**AA AGC GTC TAC GCT GCA GTC C	
INFAM A-sense2	**T**AA AGC GTC TAC GCT GCA GTC C	
INFAM- probe3	TTT GTK TTC ACG CTC ACC GTG CC	Fam-EDQ

Influenza A (Patient A)	Sequence	Label
FW primer	CTTCTRACCGAGGTCGAAACGTA	
Rv primer	TCTTGTCTTTAGCCAYTCCATGAG	
Probe 1	TCAGGCCCCCTCAAAGCCGAGA	FAM-BHQ1
Probe 2	TCAGGCCCCCTCAAAGCCGAAA	FAM-BHQ1

Sub-typing PCR human A(H3) AND A(H1)pdm09

H3/H1pdm09	Sequence	Label
H1-Sw-1306F	TGG ACT TAC AAT GCC GAA CT	
H1-Sw-1423R	CAG CGG TTT CCA ATT TCC TT	
H1-Sw-1357P3	GGA CTA TCA CGA TTC AAA TGT GAA GAA CT	ATTO425-BHQ1
H3-1541F2	AAT GTG TAC AGG GAT GAA GCA TTA AAC A	
H3-1600R	TAG GAT CCA ATC TTT GTA CCC TGA CTT	
H3-1571P2	AGC TCA ACT CCY TTG ATC TGG AAC CGG	ATTO532-BHQ1

Sub-typing PCR avian influenza H5

H5-setB mix	Sequence	Label
RF 1151	GGAACTTACCAAATACTGTCAATTTATTCA	
RF 1152	CCATAAAGATAGACCAGCTACCATGA	
RF 1153	TTGCCAGTGCTAGGGAACTCGCCAC	Fam-Tamra

Nanopore whole segement Sequence primers

Uni primers	Sequence
MBTuniG-12	ACGCGTGATCAGCGAAAGCAGG
MBTuni-13	ACGCGTGATCAGTAGAAACAAGG
